# Transcriptional programs of *Pitx2* and *Tfap2a/Tfap2b* controlling lineage specification of mandibular epithelium during tooth initiation

**DOI:** 10.1371/journal.pgen.1011364

**Published:** 2024-07-25

**Authors:** Fan Shao, An-Vi Phan, Wenjie Yu, Yuwei Guo, Jamie Thompson, Carter Coppinger, Shankar R. Venugopalan, Brad A. Amendt, Eric Van Otterloo, Huojun Cao

**Affiliations:** 1 Iowa Institute for Oral Health Research, University of Iowa College of Dentistry and Dental Clinics, Iowa City, Iowa, United States of America; 2 Department of Anatomy and Cell Biology, University of Iowa Carver College of Medicine, Iowa City, Iowa, United States of America; 3 Department of Internal Medicine and Pappajohn Biomedical Institute, University of Iowa Carver College of Medicine, Iowa City, Iowa, United States of America; 4 Department of Orthodontics, University of Iowa College of Dentistry and Dental Clinics, Iowa City, Iowa, United States of America; 5 Department of Periodontics, University of Iowa College of Dentistry and Dental Clinics, Iowa City, Iowa, United States of America; 6 Division of Biostatistics and Computational Biology, University of Iowa College of Dentistry and Dental Clinics, Iowa City, Iowa, United States of America; 7 Department of Endodontics, University of Iowa College of Dentistry and Dental Clinics, Iowa City, Iowa, United States of America; Children’s Hospital of Philadelphia, UNITED STATES OF AMERICA

## Abstract

How the dorsal-ventral axis of the vertebrate jaw, particularly the position of tooth initiation site, is established remains a critical and unresolved question. Tooth development starts with the formation of the dental lamina, a localized thickened strip within the maxillary and mandibular epithelium. To identify transcriptional regulatory networks (TRN) controlling the specification of dental lamina from the naïve mandibular epithelium, we utilized Laser Microdissection coupled low-input RNA-seq (LMD-RNA-seq) to profile gene expression of different domains of the mandibular epithelium along the dorsal-ventral axis. We comprehensively identified transcription factors (TFs) and signaling pathways that are differentially expressed along mandibular epithelial domains (including the dental lamina). Specifically, we found that the TFs *Sox2* and *Tfap2 (Tfap2a/Tfap2b)* formed complimentary expression domains along the dorsal-ventral axis of the mandibular epithelium. Interestingly, both classic and novel dental lamina specific TFs—such as *Pitx2*, *Ascl5* and *Zfp536*—were found to localize near the *Sox2*:*Tfap2a/Tfap2b* interface. To explore the functional significance of these domain specific TFs, we next examined loss-of-function mouse models of these domain specific TFs, including the dental lamina specific TF, *Pitx2*, and the ventral surface ectoderm specific TFs *Tfap2a* and *Tfap2b*. We found that disruption of domain specific TFs leads to an upregulation and expansion of the alternative domain’s TRN. The importance of this cross-repression is evident by the ectopic expansion of *Pitx2* and *Sox2* positive dental lamina structure in *Tfap2a*/*Tfap2b* ectodermal double knockouts and the emergence of an ectopic tooth in the ventral surface ectoderm. Finally, we uncovered an unappreciated interface of mesenchymal SHH and WNT signaling pathways, at the site of tooth initiation, that were established by the epithelial domain specific TFs including *Pitx2* and *Tfap2a/Tfap2b*. These results uncover a previously unknown molecular mechanism involving cross-repression of domain specific TFs including *Pitx2* and *Tfap2a/Tfap2b* in patterning the dorsal-ventral axis of the mouse mandible, specifically the regulation of tooth initiation site.

## Author summary

For adults, especially in the elderly population, tooth loss is one of the most common organ failures. Tooth development starts with formation of dental lamina that have the capability to induce a tooth development program even when recombined with non-dental mesenchyme. However, how this dental lamina is specified within the continuous naïve maxillary and mandibular ectoderm, remains poorly understood. To identify genes that are critical for dental lamina formation and dorsal-ventral axis establishment, we generated transcriptome profiles of four domains along the dorsal-ventral axis of the mouse E11.5 mandibular ectoderm. We comprehensively identified domain specific and enriched transcription factors and signaling genes along dorsal-ventral axis of the mandibular epithelium. We then investigated two loss of function mouse models for domain specific transcription factors including the dental lamina specific transcription factor *Pitx2*, and the ventral surface ectoderm specific transcription factor *Tfap2a/Tfap2b*. Unexpectedly, we found a cross-repressive relationship between domain specific transcription factors. Additionally, we found this cross-repression relationship between *Pitx2* and *Tfap2a/Tfap2b* play a critical role in patterning the dorsal-ventral axis of the mouse mandible and the initiation of tooth development program. Results from this study provide a new framework for future tooth regeneration research.

## Introduction

The development of vertebrate jaws, the primary feeding apparatus, requires the precise coordination of gene regulatory programs and signaling interactions among different tissue layers [1,2]. Recent studies indicate that the cranial neural crest cells (CNCCs), which migrate into the nascent mandibular (lower jaw) and maxillary (upper jaw) prominences, are genetically poised and their fates are primarily determined by signaling from the covering epithelial cells [3–6]. The patterning of mandibular and maxillary epithelium in-turn is thought to be established by gradients of inductive signaling pathways. For example, by embryonic day 9.5 (E9.5), the mouse mandibular epithelium can be divided broadly into a few domains by the expression pattern of *Fgf8*, *Bmp4*, *Shh*, and additional signaling pathways [7–10]. Ultimately, this partitioning results in non-dental oral epithelial, dental epithelial, and surface skin epithelial program along the dorsal-ventral axis, respectively. While the expression pattern of many signaling pathways is known, it is still unclear how gradients of these signaling pathways can precisely segregate the mandibular epithelium into distinct domains.

Proper cell lineage segregation of the epithelium is critical for tooth positioning during development of the upper and lower jaws. The precise location of teeth is essential for their function. Tooth development is governed by reciprocal interactions between dental epithelium and the underlying CNCC-derived mesenchyme. It has been shown that in most species, including mice and humans, initiation of a tooth development program is marked with a localized thickening of the maxillary and mandibular epithelium. This thickening results in a ‘strip’ of dental epithelium, known as the dental lamina (DL). Importantly, the DL has the molecular capacity to induce a tooth development program, even when recombined with non-dental mesenchyme [11]. All teeth will originate within this DL ‘strip’, going through a series of developmental transitions including the placode, bud, cap, and bell stage. Previous studies have shown that FGF and BMP pathways in maxillary and mandibular epithelium antagonize each other at the site of DL formation [7,8,12–14]. Mouse knockout (KO) models of many genes expressed in the maxillary and mandibular epithelium before DL formation—including *Pitx2*, *Sox2*, *Irx1*, *Lef1*, *Tbx1*, *Fgf8*, among others—have been generated and studied. However, despite tooth development defects at later stages, all these models present with normal DL formation [14–30]. Thus, how the DL is specified within the continuous naïve maxillary and mandibular epithelium, remains a critical and unresolved question.

The recent development of low-input RNA-seq technology—such as Smart-Seq2—provides new opportunities to address these questions by allowing transcriptome profiling of small tissues [31]. In this study, we coupled Laser Microdissection (LMD) and the Smart-Seq2 protocol (termed LMD-RNA-seq in this study) to generate transcriptome profiles of four domains—including the dental lamina (DL)—of the E11.5 mouse mandibular epithelium along its dorsal-ventral axis. Leveraging these comprehensive and spatially resolved datasets, we comprehensively identified domain specific and enriched transcription factors (TFs) and signaling genes that are differentially expressed along dorsal-ventral domains of the mandibular epithelium. To validate the functional significance of these domain specific TFs in mandibular epithelium patterning and tooth initiation, we next investigated loss of function mouse models for these domain specific TFs; namely, the DL-specific TF *Pitx2[15]*, and the ventral surface ectoderm specific TF *Tfap2a*/*Tfap2b[32]*. Unexpectedly, these analyses identified a cross-repressive relationship between epithelial domain specific networks. Furthermore, we found this cross-repressive relationship of epithelial domains’ TRNs also regulates the mesenchymal SHH and WNT pathways activity at the site of tooth initiation.

In sum, these results highlight a previously unknown molecular mechanism involving cross-repression of domain specific TFs—including *Pitx2* and *Tfap2a/Tfap2b*—in patterning the dorsal-ventral axis of the mouse mandible and specifically the regulation of a tooth initiation site. As such, they provide a new framework from which to test molecular models of tooth development, congenital tooth defects, and future tooth regeneration research.

## Results

### Transcriptome profiles of mandibular epithelial domains along the dorsal-ventral axis

**Fig 1:**
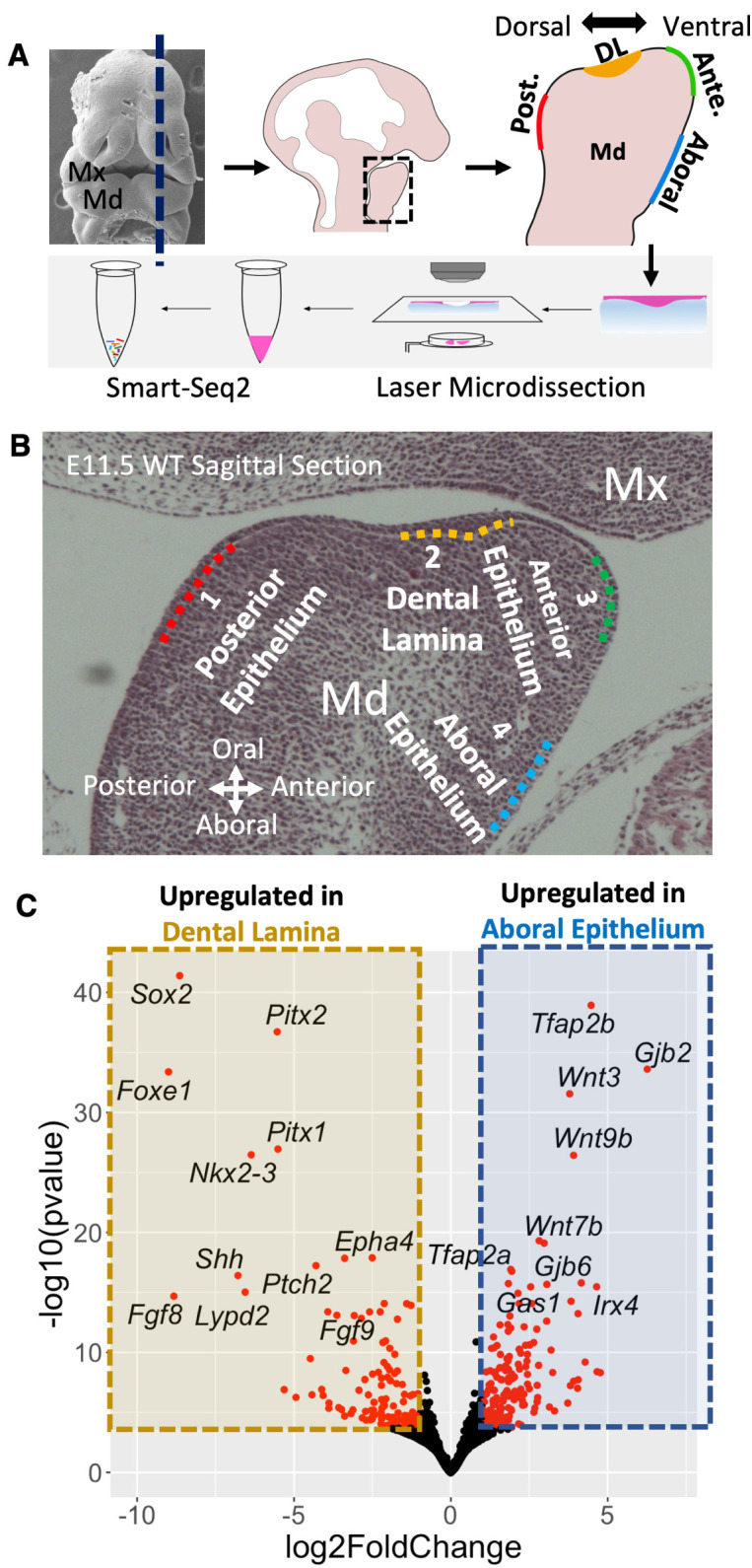
Transcriptome profiling of the E11.5 mouse mandibular epithelium along the dorsoventral axis. **A)** Workflow of Laser microdissection coupled with smart-seq2 RNA-seq (LMD-RNA-seq) protocol. **B)** LMD-RNA-seq libraries were prepared for 4 domains of mouse E11.5 mandibular epithelium along the dorsoventral axis. Dotted lines (#1–4) define the regions of epithelial cells that were collected with laser microdissection. **C)** Volcano plot showing differentially expressed genes (DEGs) of the E11.5 dental lamina (#2 domain) compared with the aboral ectoderm (#4 domain). Red dots indicate DEGs with a absolute log2 fold-change larger than 1.0 and a adjusted p value less than 0.01. Abbreviations: DL, dental lamina; Md, Mandible; Mx, Maxilla.

To identify genes that are critical for mandibular epithelial patterning, we first applied Laser Microdissection (LMD) coupled Smart-Seq2 RNA-seq [31] (LMD-RNA-seq) to generate transcriptome profiles of four domains along the dorsal-ventral axis of the E11.5 mouse mandibular epithelium (also known as the oral-aboral axis, we will refer to it as the dorsal-ventral axis throughout this study, **Fig 1A**). The four domains, from dorsal to ventral, included: 1) a posterior/dorsal domain, located posterior to the dental lamina (DL) that will develop into non-dental oral epithelium; 2) a DL domain, developing into dental epithelium; 3) an anterior domain, located anterior to the DL and developing into surface skin epithelium; and, 4) an aboral/ventral domain, also developing into surface skin epithelium (**Fig 1B**). We performed differentially expressed gene (DEG) analysis between the transcriptome profiles of these four domains with DESeq2[33] (**Figs 1C** and **S1**). To control for variability associated with lowly expressed genes, we further applied the ASHR algorithm [34] to estimate shrunken (conservative) log2 fold changes.

During initial analysis, we found that the anterior domain epithelium and aboral domain epithelium are molecularly similar (**S1E Fig**). Therefore, in the subsequent analysis, we focused on three domains: the posterior domain, the DL domain, and the aboral domain. Pair-wise DEG analysis comprehensively identified two classes of genes that we termed, “domain specific” or “domain enriched”. Specifically, we defined domain specific genes as genes that were significantly upregulated in one domain relative to the other two domains. In contrast, we defined domain enriched genes as genes that were significantly upregulated in one domain relative to one other domain. For example, *Pitx2* is a domain specific gene for the DL domain; *Sox2* is a domain enriched gene for both the posterior domain and the DL domain. Through this analysis, we defined 36 posterior domain specific genes, 36 DL specific genes; and 76 aboral domain specific genes. Similarly, we identified 175 posterior domain enriched genes, 848 DL enriched genes, and 239 aboral domain enriched genes (**S1 Table**).

**Fig 2:**
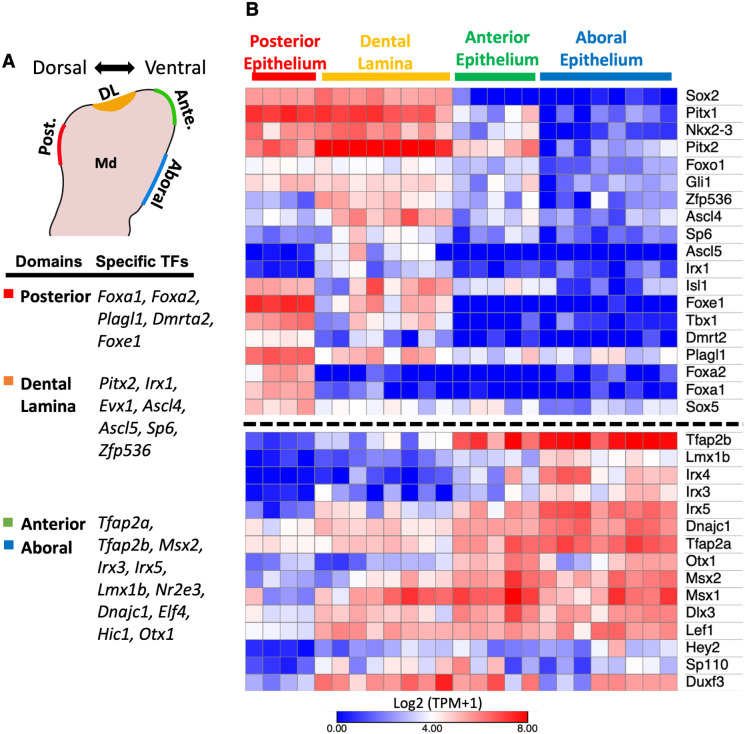
Domain specific and domain enriched transcription factors along the mandibular epithelial dorsoventral axis. **A)** List of domain specific transcription factors associated with the 4 domains profiled along the dorsoventral axis of the mouse E11.5 mandibular epithelium. **B)** Expression heatmap of domain specific and domain enriched transcription factors, including those listed in panel A, along the dorsoventral axis of the E11.5 mandibular epithelium. Rows represent genes, columns represent samples. Dorsal enriched genes are shown in top panel while ventral enriched genes are shown in bottom panel. log2(TPM+1) expression values were used in the heatmap. Abbreviation: TPM, Transcript Per Million.

Closer examination of domain specific and domain enriched genes revealed that a large number encoded transcription factor (TF) and signaling pathway proteins. For example, DEGs analysis comparing the DL with the aboral epithelium identified significant enrichment for *Sox2*, *Pitx2*, *Foxe1*, *Pitx1*, *Nkx2-3*, *Shh*, and *Fgf8* within the DL and *Tfap2b*, *Gjb2*, *Wnt3*, *Wnt9b*, *Wnt7b*, *Tfap2a*, and *Irx4* within the aboral epithelium (**Fig 1C**). To focus our analysis on only domain specific or enriched TFs, we next intersected the identified DEGs with all known mouse TFs listed in the AnimalTFDB database [35]. We identified the following domain specific TFs in the E11.5 mandibular epithelium: *Foxa1*, *Foxa2*, *Plagl1*, *Dmrta2*, and *Foxe1* defined the posterior epithelium; *Pitx2*, *Irx1*, *Evx1*, *Ascl4*, *Ascl5*, *Sp6*, and *Zfp536* defined the DL; *Tfap2a*, *Tfap2b*, *Msx2*, *Irx3*, *Irx4*, *Irx5*, *Lmx1b*, *Nr2e3*, *Dnajc1*, *Elf4*, *Hic1*, and *Otx1* defined the aboral epithelium (**Fig 2A**). We generated an expression heatmap to visualize expression of domain specific and enriched TFs in different domains of mandibular epithelium (**Figs 2B** and **S2A**). The heatmap revealed that the anterior epithelium and aboral epithelium expressed a large set of shared TFs, including *Tfap2a*, *Tfap2b*, *Msx1*, *Msx2*, *Dlx3*, and *Lef1*. Similarly, the posterior epithelium and DL shared expression of some TFs including *Sox2*, *Nkx2-3*, *Pitx1*, *Isl1*, and *Tbx1*. Some of the DL specific or enriched TFs (e.g., *Pitx2*, *Sox2*, *Tbx1*, etc.) are known to have critical roles during tooth development [15,16,26,28,36–41], while for others TFs (e.g., *Foxe1*, *Nkx2-3*, *Klf5*, *Zfp536*, *Ascl5*, etc.), their function, specifically during mandibular epithelium patterning and DL formation, is unclear.

**Fig 3:**
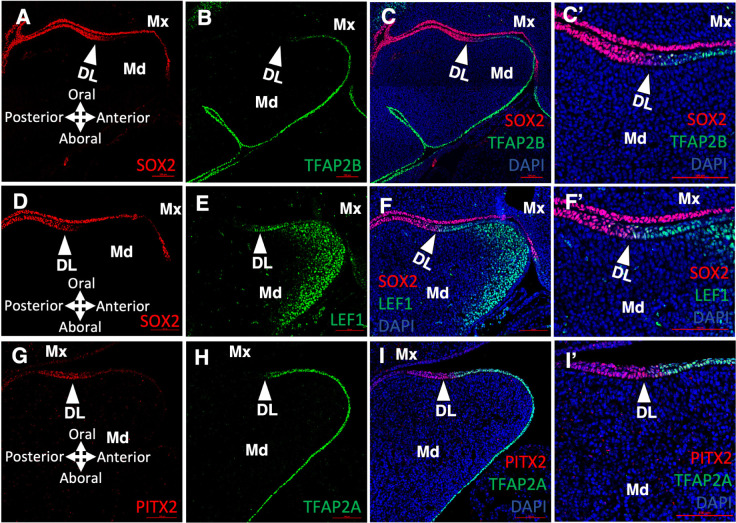
Complementary expression patterns of domain specific transcription factors. **A, B, C**) SOX2, TFAP2B and merged immunofluorescent (IF) staining of an E11.5 mouse head sagittal section. **C’**: higher magnification of the dental lamina region in panel **C**. **D, E, F**) SOX2, LEF1 and merged IF staining of an E11.5 mouse head sagittal section. **F’**: higher magnification of dental lamina region in panel **F**. **G, H, I**) PITX2, TFAP2A and merged IF staining of an E11.5 mouse head sagittal section. **I’**: higher magnification of dental lamina region of **I**. White arrowheads in all panels point to the dental lamina. Abbreviations: DAPI, DAPI nuclear counterstain; DL, dental lamina; Md, Mandible; Mx, Maxilla. Scale bar = 100 μm.

Finally, to verify the expression pattern of these domain specific or enriched TFs in situ, we utilized immunofluorescent staining (IF) of a subset of TFs to directly visualize expression along the dorsal-ventral axis of the mandibular epithelium at E9.5 and E11.5 (**Figs 3** and **S3**). Interestingly, we found that SOX2 and TFAP2A/TFAP2B expression broadly divided the mandibular epithelium into two complementary domains along the dorsal-ventral axis. Specifically, at E11.5, SOX2 expression covered the dorsal side of the mandibular epithelium, and up to the posterior part of the DL, with expression reduced in the anterior DL and ceasing further ventral (**Fig 3A, 3C, 3D and 3F**). In contrast, at E11.5, TFAP2A/TFAP2B expression, while being high throughout the ventral side of the mandibular epithelium, was dramatically reduced after reaching SOX2 positive cells in the DL (**Fig 3B and 3H)**. Further, PITX2 and LEF1 were expressed at the interface of SOX2 and TFAP2A/TFAP2B domains, which in part, coincided with the DL (**Fig 3**). Specifically, while PITX2 expression covered the entire DL (**Fig 3G and 3I)**, LEF1 expression was restricted to the anterior part of the DL (**Figs 3E–3F, and S3J–S3L**). Interestingly, at E9.5—and in contrast to E11.5—the expression pattern of SOX2, TFAP2A, TFAP2B and LEF1 had broader overlapping regions within the “naïve” mandibular epithelium (**S3 Fig**). These findings are consistent with a progressive restriction, or enrichment of expression, of these domain specific or enriched TFs. Indeed, it has been shown that *Pitx2* expression is initially broad in the oral cavity [12], contributing to cells eventually found in the non-dental and facial skin epithelium [15], but later becomes highly restricted to the dental epithelium. We found protein expression pattern detected by IF staining (**Figs 3** and **S3**) agreed with mRNA expression detected by LMD-RNA-seq (**Fig 2**). Additionally, mRNA expression pattern detected by In-situ hybridization (ISH) of selected TFs (*Pitx2* and *Lef1*, **S3M–S3O Fig**) shows similar expression pattern detected by IF staining (**S3J–S3L Fig**).

### Correlation between domain specific transcription factors and signaling pathways

In addition to domain specific or enriched TFs, LMD-RNA-seq analyses also identified many signaling pathway genes that correlated with TF-expression patterns. Using LMD-RNA-seq data (**Figs 1** and **S1**) and the KEGG pathway database [42], we constructed a domain expression heatmap of genes belonging to known signaling pathways (**S4 Fig**). Doing such, we identified that expression of dorsal domain TFs (e.g., *Sox2*, *Foxe1*, *Foxa1*, and *Foxa2*) correlated with several genes of the SHH-signaling pathway (**S4A Fig**). For example, consistent with previous studies [10,43,44], *Shh* expression was restricted to the dorsal side—including the DL—and absent from the ventral side of the E11.5 mandibular epithelium. Additionally, SHH-associated receptors, *Ptch1* and *Ptch2*, and downstream TF, *Gli1*, were also enriched on the dorsal side of the mandibular epithelium. Interestingly, expression of *Gas1*, a negative regulator of the SHH pathway, was restricted to the ventral side. In contrast to SHH-pathway components, we identified that expression of ventral domain TFs (e.g., *Tfap2a*, *Tfap2b*, *Msx1*, and *Irx4*) correlated with several genes of the WNT-signaling pathway (**S4B and S4C Fig**). For example, the majority of WNT ligands (e.g., *Wnt3a*, *Wnt3*, *Wnt7a*, *Wnt9b*, *Wnt10a* and *Wnt10b*) was restricted to the aboral/ventral side of the E11.5 mandibular epithelium. Even the few WNT ligands whose expression extended more posteriorly (e.g., *Wnt6*, *Wnt4*, *Wnt7b* and *Wnt5a*) had higher expression in ventral epithelial domains. Interestingly, in addition to ligands expression, we also found high expression of several negative regulators of the WNT pathway (e.g., *Axin2*, *Kremen2*, *Sostdc1*, and *Ctnnd2[45]*) in ventral domain epithelium. Accordingly, assessemnt of WNT pathway activity via α-LEF1 IF, revealed the highest epithelial enrichment in the anterior part of the DL with a gradual diminishment moving towards ventral epithelium (**Fig 3E and 3F**). However, in contrast to the ventral epithelium, α-LEF1 signal was highly enriched in the underlying ventral mesenchyme, consistent with the secreted WNT ligands from ventral epithlium inducing WNT pathway activity in the underlying mesenchyme (**Fig 3E and 3F)**. Given that constitutive activation of the WNT pathway in oral and dental epithelium leads to supernumary teeth formation [46–49], we suspect that the negative regulators of the WNT pathway prevent the dental lineage fate in ventral domain epithelium. Interestingly, at E11.5, we found expression of genes indicative of SHH (e.g., *Ptch1*, *Ptch2* and *Gli1*) and WNT (e.g., *Lef1* and *Axin2*) pathway activity converged at the site of DL formation (**S4 Fig**). Finally, in addition to SHH and WNT signaling, we found expression of multiple genes in the FGF and BMP pathway that were restricted or enriched in the dental epithelium (e.g., *Fgf20*, *Bmp2*, *Fgf9*, *Fgf8*, and *Id4*) (**S4D Fig**). In sum, similar to TFs, we found complementary expression domains of signaling pathways along the dorsal-ventral axis of the mandibular epithelium, typified by SHH dorsally and WNT ventrally.

### Loss-of-function models provide evidence of a mutually restrictive relationship between DL and aboral domain networks

**Fig 4:**
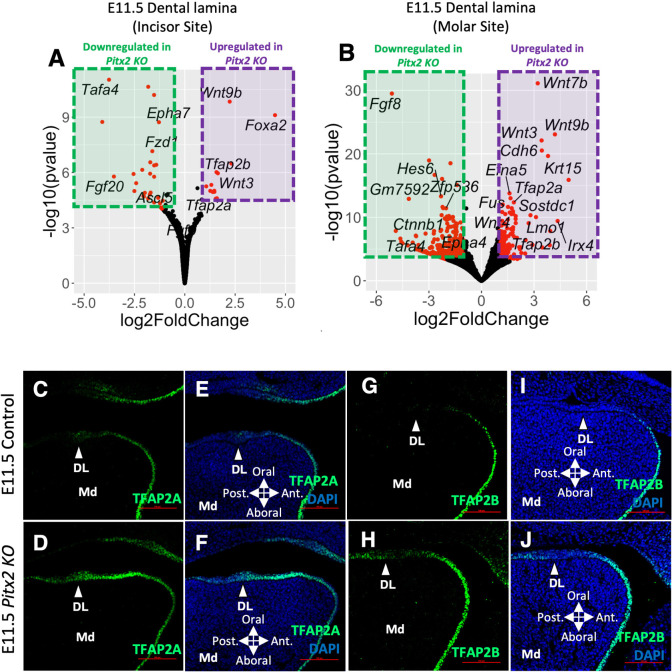
Upregulation of aboral domain specific or enriched genes in the dental lamina of the *Pitx2* KO. **A, B)** Volcano plots of DEGs comparing the dental lamina at the mesial/incisor site (A) or the distal/molar site (B) of E11.5 *Pitx2 KOs* with littermate controls. Red dots indicate DEGs with an absolute log2 fold-change larger than 1.0 and a adjusted p value less than 0.01. **C-J)** TFAP2A (C-F) or TFAP2B (G-J) immunofluorescent (IF) staining of an E11.5 mouse head sagittal section in a control (C, E, G, I) or *Pitx2* KO (D, F, H, J). Note, panels E, F, I, and J are the same section as in panels C, D, G, and H, respectively, but include visualization of the DAPI counterstain. White arrowheads in all panels point to the dental lamina. Abbreviations: DAPI, DAPI nuclear counterstain; DL, dental lamina; Md, Mandible. Scale bar = 100 μm.

To determine the role of domain specific TFs in mandibular epithelium patterning and DL formation, we next examined two loss-of-function mouse models. First, we examined gene expression changes associated with loss of *Pitx2*, one of the earliest and most specific markers for the dental epithelium. Here, we utilized a *Pitx2* ‘knockout’ (*Pitx2 KO*) allele *[15]*, which harbors an IRES-Cre knock-in cassette at the *Pitx2* locus, preventing protein production. Previous studies have shown that loss of *Pitx2* doesn’t affect dental lamina formation but causes tooth development arrest at placode (maxillary teeth) or bud (mandibular teeth) stage with full penetrance [15,16,30,36,50,51]. Consistent with previous reports, we found the localized thickening DL strip formation is presented in *Pitx2 KO* mice at E11.5 (**Figs 4 and S5A–S5F**). To assess DL specific gene expression changes upon loss of *Pitx2*, we next used LMD-RNA-seq to profile the E11.5 DL from both *Pitx2 KOs* and littermate controls. In both incisor and molar sites, we found that ventral domain specific TFs—including *Tfap2a* and *Tfap2b*—were among the top upregulated genes in *Pitx2 KOs*, while DL specific TFs—including *Ascl5* and *Zfp536*—were among the top downregulated genes (**Fig 4A and 4B**). Additional ventral domain specific or enriched genes, including WNT ligands (*Wnt9b*, *Wnt3*, *Wnt7b*, and *Wnt4*) and a WNT inhibitor (*Sostdc1*), were also significantly upregulated in the DL of *Pitx2 KOs*. Conversely, DL specific or enriched genes, including FGF ligands (*Fgf8* and *Fgf20*) and EPH receptors (*Epha7* and *Epha4*), were significantly downregulated in *Pitx2 KOs*. Reduction of Fgf8 in the DL of *Pitx2* KOs has been reported in previous studies [15,16,36]. IF staining confirmed that ventral domain specific TF expression, including that of TFAP2A and TFAP2B, was upregulated and expanded dorsally into the DL of *Pitx2 KOs* (**Fig 4C–4J**). Thus, loss of a dental epithelium specific TF correlated with a gain of ventral domain specific genes’ expression.

**Fig 5:**
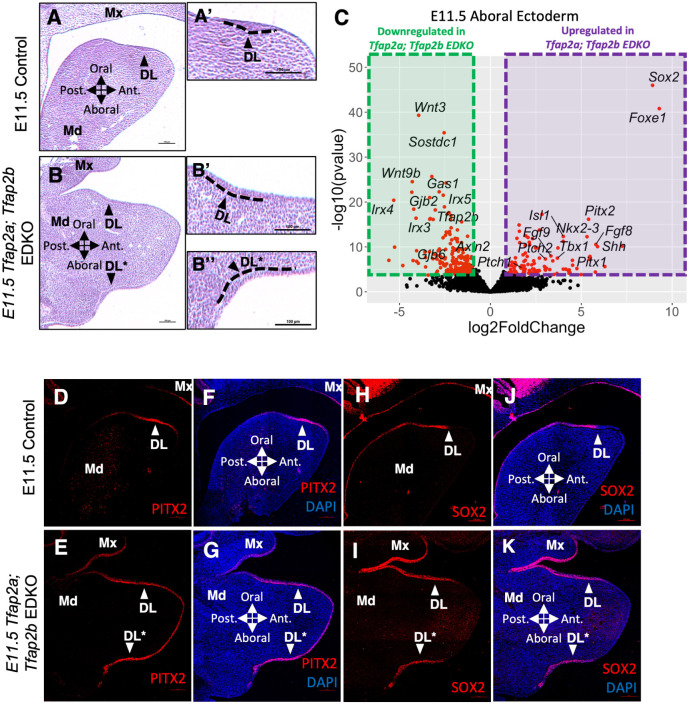
Upregulation of dental lamina specific or enriched genes in the aboral domain of the *Tfap2a; Tfap2b EDKO*. **A, B)** H&E staining of a sagittal section of an E11.5 control and *Tfap2a; Tfap2b EDKO* head. Black arrowheads point to the multilayered dental lamina, including the ectopic multilayered epithelium found in the aboral domain of the *Tfap2a; Tfap2b EDKO* in panel B. A’, B’, B”: Higher magnification of dental lamina and ectopic dental lamina in panel A and B. **C)** Volcano plot of DEGs comparing the aboral epithelium of E11.5 *Tfap2a; Tfap2b EDKOs* with littermate controls. Red dots indicate DEGs with a absolute log2 fold-change larger than 1.0 and a adjusted p value less than 0.01. **D-K)** PITX2 (D-G) or SOX2 (H-K) immunofluorescent (IF) staining of an E11.5 mouse head sagittal section in a control (D, F, H, J) or *Tfap2a; Tfap2b EDKO* (E, G, I, K). Note, panels F, G, J, and K are the same section as panels D, E, H, and I, respectively, but include visualization of the DAPI counterstain. Original and ectopic dental lamina are marked by white arrowheads. Abbreviations: DAPI, DAPI nuclear counterstain; DL, dental lamina; DL*, ectopic dental lamina; Md, Mandible; Mx, Maxilla. Scale bar = 100 μm.

Next, we examined the early ectodermal (using the CRECT Cre mouse line, driven by a *Tfap2a* ectodermal specific enhancer [52], which active by ~ E7.5) double knockout of *Tfap2a* and *Tfap2b* mouse model (referred to as *Tfap2a; Tfap2b EDKO* in this study) to determine the effect of removal of an ventral domain specific TFs. Previous analyses of this loss-of-function model found an ectopic incisor (EI) forming on the aboral/ventral side of the mandible [32]. Given that previous analyses of *Tfap2a; Tfap2b EDKOs* did not include early stages of odontogenesis (e.g., DL formation), we first examined early stages of tooth development in mutants versus controls by histology (**Figs 5** and **S5G–S5K**). We found, at E11.5, an epithelium characteristic of the DL (e.g., thickened multilayer epithelium) expanded ventrally in *Tfap2a; Tfap2b EDKOs* (**Fig 5A and 5B**). Subsequently, at E12.5, an ectopic tooth placode formed at the bottom of the aboral/ventral side of the mandible (separated from the normal incisor (I_1_) placode) in *Tfap2a; Tfap2b EDKOs* (**S5H and S5H’ Fig**). Consistent with the previous findings [32], late stage (E14.5-E18.5) H&E and skeletal preparations revealed that in most *Tfap2a; Tfap2b EDKOs*, this ectopic placode eventually developed into an aboral ectopic incisor. While the ectopic incisor grew with grossly normal morphology and size, the lingual-labial axis of this ectopic incisor was inverted (**S5I–S5K** and **S6 Figs)**. Thus, histology suggested that in *Tfap2a; Tfap2b EDKOs*, the ventral domain of the mandibular epithelium, which normally commits to the surface skin lineage, is converted into dental epithelium lineage.

To determine the molecular level changes in *Tfap2a; Tfap2b EDKOs*, we generated transcriptome profiles of E11.5 ventral epithelium in *Tfap2a; Tfap2b EDKOs* and littermate controls using LMD-RNA-seq. DEG analysis of RNA-seq data revealed that DL specific or enriched TFs—including *Sox2*, *Foxe1*, *Pitx2*, *Pitx1*, *Nkx2-3*, *Tbx1* and *Isl1*—were among the top upregulated genes in the ventral epithelium of *Tfap2a; Tfap2b EDKOs* (**Fig 5C**). In contrast, ventral epithelium specific or enriched TFs—including *Irx4*, *Irx3* and *Irx5*—were among the top downregulated genes in the *Tfap2a; Tfap2b EDKOs* (**Fig 5C**). Along with changes of domain specific or enriched TF expression, we also found upregulation of FGF and SHH pathway genes (e.g., *Fgf8*, *Fgf9*, *Shh*, and *Ptch2*) and downregulation of WNT pathway genes (e.g., *Wnt3*, *Sostdc1*, and *Wnt9b*) in the ventral epithelium of *Tfap2a; Tfap2b EDKOs* (**Fig 5C**). Consistent with LMD-RNA-seq analysis, IF staining of two key DL TFs, PITX2 and SOX2, confirmed their ectopic and ventrally expanded expression in the ventral epithelium of *Tfap2a; Tfap2b EDKOs* (**Fig 5D–5K**). However, this ventrally expansion of DL specific/enriched genes expression was not observed in either single KOs (**S7 Fig**). In summary, loss of ventral domain epithelial specific TFs correlated with a gain of DL domain specific genes’ expression.

LMD-RNA-seq analysis of epithelial tissue from *Pitx2 KOs* and *Tfap2a; Tfap2b EDKOs* identified that loss of domain specific TFs correlated with upregulation of genetic program defining the reciprocal domain. To further test the nature of the potential cross-repression between PITX2 and TFAP2A/TFAP2B in a more homogenous, tractable system, we utilized a human oral epithelial cell line, GMSM-K cells [53]. Note, GMSM-K cells have robust *TFAP2A* expression but very low *TFAP2B* expression (personal communication with Robert Cornell, based on RNA-seq data). First, to determine PITX2’s impact on TFAP2A expression, we transfected GMSM-K cells with either a GFP or PITX2 encoding construct and monitored TFAP2A expression. Compared to un-transfected or GFP transfected control cells, PITX2 transfected cells had a significant reduction in TFAP2A mRNA and protein levels (**S8A–S8B Fig**). Secondly, to determine TFAP2’s impact on PITX2’s activity, we utilized an in vitro luciferase reporter assay comprised of the *Pitx2* promoter driving luciferase expression. Given PITX2 can autoregulate its own promoter [26,28,40], transfection of GMSM-K cells with both the *Pitx2* promoter reporter and the PITX2 encoding construct resulted in a significant increase in luciferase activity. However, this activation was significantly decreased in a dose responsive manner upon co-transfection with *Tfap2a* or *Tfap2b* (**S8C Fig**). Collectively, these data highlight the cross-repressive relationship between PITX2 and TFAP2 paralogs.

### Communication between epithelium and mesenchyme defines the tooth initiation site and the dorsal-ventral axis of the mandible

The development of teeth is governed by reciprocal interactions between dental epithelium and the underlying neural crest derived mesenchyme. While previous tissue recombination studies have shown that the tooth initiation capacity first resides in the dental epithelium [54–56], odontogenic competent mesenchyme is also a prerequisite for the tooth development [57]. Having delineated the cross-repressive nature of core networks in the overlying mandibular epithelium, we next examined how these networks impinge upon the underlying mesenchyme in both wild-type and loss-of-function models.

First, we applied LMD-RNA-seq in WT embryos to understand positional gene expression patterns in the dental mesenchyme during normal tooth initiation. Specifically, we partitioned the E11.5 WT dental mesenchyme into three parts: lingual/posterior, middle (right under the DL) and labial/anterior mesenchyme (**S9A Fig**). We found *Foxf1* and *Foxf2*, known SHH downstream readouts [58], were enriched in lingual mesenchyme, while *Msx1*, *Msx2*, and *Lef1*, known BMP and WNT readouts [57,59,60], were enriched in labial mesenchyme (**S9A Fig**). IF analysis of FOXF1 and LEF1 revealed two complementary domains (e.g., FOXF1 in dorsal part, LEF1 in ventral part) in the E10.5 mandibular mesenchyme (**S9B Fig**). Moreover, at E11.5, this FOXF1-LEF1 (i.e., SHH-WNT pathway activity) interface persisted and was positioned directly below the DL, with a few cells in the dental mesenchyme expressing both genes (**S10A–S10C Fig**). This complementary SHH-WNT pathway activity pattern is consistent with the expression of SHH and WNT pathway ligands in the overlying epithelium.

**Fig 6:**
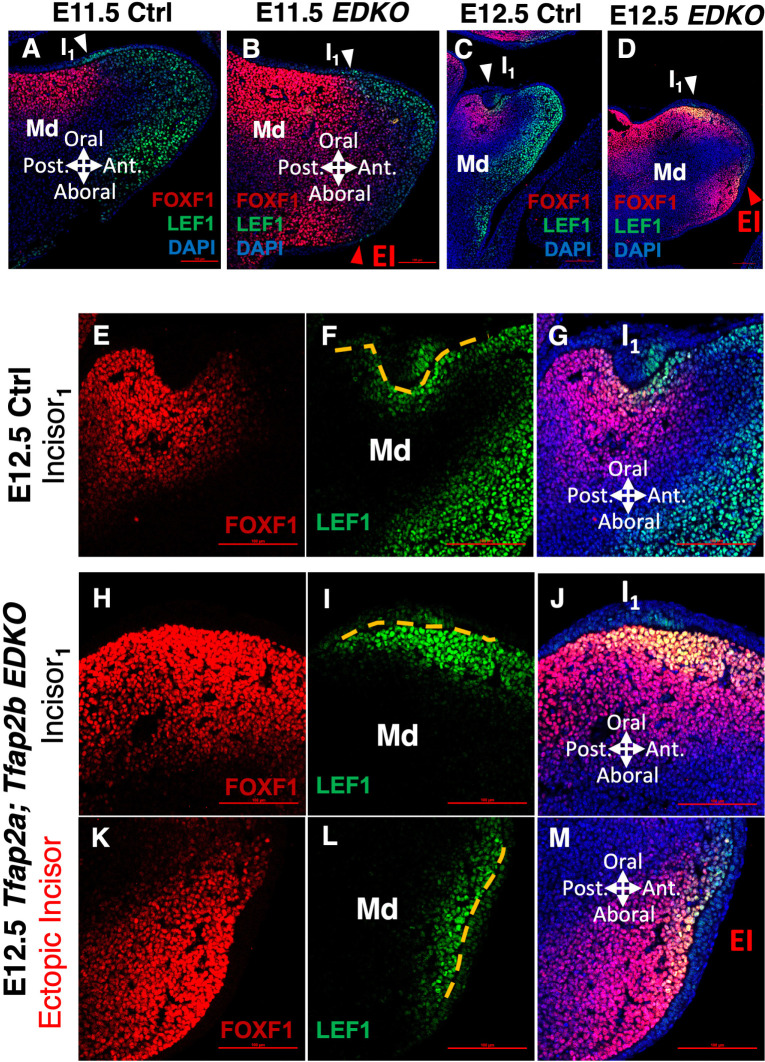
Both original and ectopic tooth initiation sites correlate with a mesenchymal SHH-WNT interface. **A-D)** IF staining of FOXF1 (red, a readout of SHH pathway activity) and LEF1 (green, a readout of WNT pathway activity) in an E11.5 (A, B) or E12.5 (C, D) control (A, C) or *Tfap2a; Tfap2b EDKO* (B, D) mouse head sagittal section. The white arrowhead in panels A-D points to the mesenchymal SHH-WNT pathway interface at the site of original incisor formation. The red arrowhead in panels B and D points to the mesenchymal SHH-WNT pathway interface at the site of ectopic incisor formation. **E-M**) Higher magnification of the site of original incisor formation (E-G) in panel C or of the site of original (H-J) or ectopic (K-M) incisor formation in panel D. Yellow dashed line in panels F, I, and L labels the relative position of the forming dental placode. Abbreviations: DAPI, DAPI nuclear counterstain; EI, ectopic incisor; I1, original incisor; Md: mandible. Scale bar = 100 μm.

Second, to examine how these mesenchymal domains were impacted upon perturbation of epithelial programs, we next examined SHH and WNT pathway activity in the mesenchyme of *Pitx2 KOs* and *Tfap2a; Tfap2b EDKOs* by IF for FOXF1 and LEF1. In E11.5 *Pitx2 KOs*—associated with an upregulation of TFAP2A/TFAP2B and WNT ligands in the DL (**Fig 4**)—we found dorsal expansion and upregulation of LEF1 and CTNNB1 (β-catenin), along with a reduction of FOXF1, on the posterior side of the dental mesenchyme (**S10D–S10F, and S11C and S11D Figs**, arrows in **S10F’** and **S11D’ Figs**), relative to controls (**S10A–S10C, and S11A and S11B Figs**). Interestingly, we found no significant changes in *Shh* expression in *Pitx2 KOs*, measured by LMD-RNA-seq. In contrast to *Pitx2 KOs*, in E11.5 *Tfap2a; Tfap2b EDKOs*—associated with a reduction of WNT ligands in the ventral domain epithelium (**Fig 5**)—we found a reduction of LEF1 in the mandibular mesenchyme (**Fig 6B**), relative to controls (**Fig 6A**). Moreover, the LEF1 reduction was associated with an ventral expansion of FOXF1 throughout the E11.5 *Tfap2a; Tfap2b EDKO* mandibular mesenchyme (**Fig 6B**), in contrast to FOXF1’s dorsal restriction in controls (**Fig 6A**). This ectopic expansion of FOXF1 correlated with the ectopic expansion of SHH-pathway components, *Shh*, *Ptch1*, and *Gli1* in the ventral domain epithelium of *Tfap2a; Tfap2b EDKOs* (**Fig 5C**). Thus, epithelial disruption of signaling pathways in loss-of-function models was mirrored by corresponding changes in the mandibular mesenchyme.

Finally, we leveraged our *Tfap2a; Tfap2b EDKO* model—which presented with the ectopic ventral incisor—to further decipher the importance of these networks in defining tooth placement. Interestingly, despite the broad expansion of PITX2 and SOX2 expression in mutants (**Fig 5**), we found the ectopic incisor was always located at the bottom of the ventral/aboral side of the mandible (**S5G–S5K** and **S6 Figs**). Given the correlation of a SHH-WNT interface at the site of wild-type incisor formation, we predicted that such an interface could define the placement of the ectopic incisor. Because we observed a slight developmental delay of the ectopic incisor in *Tfap2a; Tfap2b EDKOs*, relative to the normal incisor, we examined FOXF1 and LEF1 expression at E12.5 when the site of ectopic incisor is clearly identifiable. Similar to E11.5 controls (**S10A–S10C Fig**), in E12.5 controls, FOXF1 and LEF1 expression formed a converging interface underneath the incisor placode (**Fig 6C and 6G**). Consistent with functional significance of the SHH-WNT interface, in *Tfap2a; Tfap2b EDKOs* we found a convergence of FOXF1 and LEF1 expression at not only the ‘original’ incisor site (**Fig 6H–6J**), but the ectopic incisor site as well (**Fig 6K–6M**). Interestingly, compared to the ‘original’ incisor, the orientation of the FOXF1-LEF1 interface was reversed at the site of the ectopic incisor placode (**Fig 6D, 6J and 6M**), consistent with the inverted lingual-labial axis of the ectopic incisor (**S6 Fig**) [61].

Collectively, these results suggest that in *Tfap2a; Tfap2b EDKOs* the ventral mandibular mesenchyme is converted to a mirrored image of dorsal mesenchyme. Thus, epithelial signaling to the mesenchyme helps establish the dorsal-ventral axis of the mandible. Further, this communication—particularly between SHH and WNT pathways—correlates with the location, and orientation, of tooth development. While correlative, these findings suggest functional significance of this interface in tooth development, consistent with previous reports of their individual roles in such [8,10,58,62–64].

## Discussion

**Fig 7:**
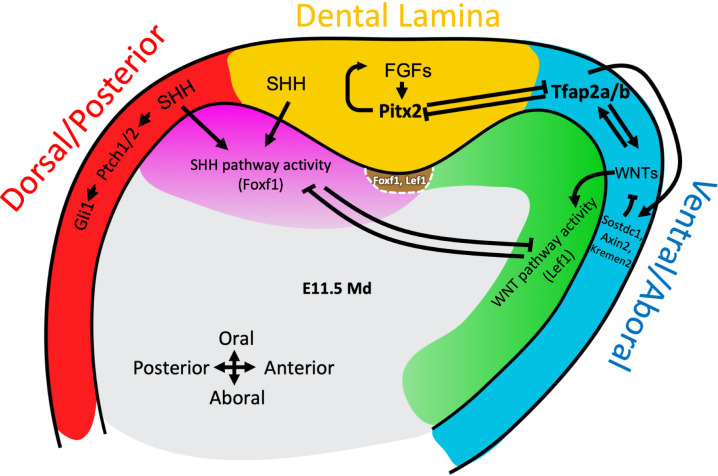
Working model for establishment of the mandibular dorsoventral axis and dental lamina placement. Gradients of inductive signals—including SHH, FGF, BMP and WNT—establish broad domains of mandibular epithelium by induction of transcription factor expression. Domain specific transcription factors—including PITX2 and TFAP2A/TFAP2B—inhibit each other, refining epithelial domains. Further, domain specific transcription factors feedback on expression of signaling pathway genes. Cumulatively, refined signaling pathways communicate with the underlying mesenchyme to establish the mandibular dorsoventral axis and the site of tooth initiation.

In this study, through LMD-RNA-seq, we comprehensively profiled gene expression pattern for different regions of the developing mandible during dental lamina formation. We uncovered a previously unknown molecular mechanism establishing the dorsal-ventral axis of the mouse mandible, which in turn specifies the site of tooth initiation (**Fig 7**). We identified groups of ‘domain specific’ and ‘domain enriched’ transcription factors and signaling pathway genes that play major roles in coordinating these processes. Specifically, our results identified that *Tfap2a* and *Tfap2b* establish the ventral domain epithelium through promoting expression of WNT ligands, resulting in WNT pathway activation in ventral mesenchyme. In contrast, *Shh* expression is enriched within the dorsal domain epithelium, stimulating SHH activity in the dorsal mesenchyme. Several previous studies have shown the importance of SHH and WNT pathways in tooth development, including: 1) triple KO of *foxf1; foxf2a; foxf2b* in zebrafish leads to a complete absence of tooth buds [58]; 2) conditional KO of SHH signaling in the mesenchyme leads to disruption of the mandibular oral-aboral axis [10]; 3) double KO of *Msx1* and *Msx2* leads to tooth development arrest at the DL to placode stage [8,62]; 4) conditional KO of β-catenin (i.e., removal of WNT pathway activity) in the dental mesenchyme leads to tooth development arrest at the cap stage [63] while excessive WNT activity in the mesenchyme also inhibits tooth formation [64]; 5) Constitutively activation of the WNT signaling pathway in dental and oral epithelium (e.g., *K14-CreER; Apc cKO* model) leads to supernumerary teeth formation [46–49], while over-expression of Lef1 in dental epithelium leads to active cell proliferation and an enlarged & branching incisor’s stem cell compartment [40].

At the interface of SHH and WNT pathways, *Pitx2* expression establishes the dental epithelium, in part through forming feedback loop with FGF ligands expression. Further, we identified cross-repression between the domain specific transcription factors *Pitx2* and *Tfap2a/Tfap2b*, which provides molecular explanation for the previously reported tooth development phenotypes: early tooth development arrest in *Pitx2 KO* mice [15] and ectopic tooth formation in *Tfap2a; Tfap2b EDKO* mice [32]. We speculate that cross-repression between domain specific transcription factors provides a mechanism to translate broad domains—established by gradients of inductive signal pathways including SHH, FGF, BMP and WNT—into precisely segregated domains within the mandibular epithelium (**Fig 7**).

### Genetic redundancy provided by transcription factor paralogs

While our study identified dorsoventral localized ‘domain specific’ and ‘domain enriched’ TFs within the mandibular epithelium, another key observation in this study was the common co-expression of TF paralogs within domains. For example, *Foxa1*/*Foxa2*, *Pitx1*/*Pitx2*, *Irx3*/*Irx4*/*Irx5*, *Msx1*/*Msx2*, and *Tfap2a*/*Tfap2b* have similar, although slightly different, expression pattern in the mandibular epithelium. Along with the technical limitations of previous epithelial CRE lines (i.e., not expressed early enough to target ‘pre-dental lamina’ epithelium), we speculate that the genetic redundancy of co-expressed paralogs has likely contributed to the paucity of loss-of-function models with altered dental lamina formation and disruption of the dorsal-ventral axis of the mouse mandible. Indeed, paralog redundancy may provide robust developmental buffering from the misplacement of teeth—an outcome having potentially devastating consequences in the context of evolutionary survival of jawed vertebrates. The importance of redundancy is specifically highlighted by our *Tfap2a; Tfap2b EDKO* model. For example, while epithelial loss of either *Tfap2a* or *Tfap2b* alone did not impact dental lamina placement or gross tooth development, only with their simultaneous loss did an aboral, ectopic dental lamina and incisor form [32]. Similar studies have yet to be completed for paralogs expressed in other domains and thus the generation of double, or potentially triple, knockout mouse models may be necessary to uncover additional details of these very early steps of dental lamina development. For example, while *Pitx2*—arguable one of the most well-known dental lamina markers—mutants display tooth bud arrest, the relative development and placement of the dental lamina occurs normally [16]. Likewise, loss of *Pitx1*, although associated with upper and lower jaw defects, does not grossly impact early tooth development [14,65]. While *Pitx1*/*Pitx2* compound mutants have been generated, an assessment of dental lamina development has yet to be completed, in part, owing to the early embryonic lethality associated with loss of both paralogs [66]. Thus, conditional approaches targeting the epithelium alone, such as we have conducted for *Tfap2a* and *Tfap2b*, may be necessary to fully elucidate these networks.

### Limitations of current study and considerations for future studies

While our study has uncovered a previously unrecognized competitive intersection of signaling pathways and transcriptional networks driving dental lamina placement and establishing the dorsal-ventral axis of the mouse mandible, several important questions remain. For example, why—despite the shared overlap of several domain specific TFs in dorsal-ventral domains of the incisor and molar epithelium, including aboral expression of *Tfap2a* and *Tfap2b*—do *Tfap2a; Tfap2b EDKOs* not display ectopic molars? While it is possible that the temporal and spatial expression—and thus functional activity of CRE—driven by the ectodermal CRE, CRECT [52], may account for differences in incisor-molar phenotypic outcomes upon recombination, it is also interesting to note that *Tfap2c*, a third TFAP2 paralog, is also expressed at low levels, potentially providing additional compensation at the site of the molar. Equally plausible is that although core gene sets are shared between incisor and molar sites, enough divergence in networks exist that phenotypic outcomes are distinct between the two sites. Indeed, previous studies have shown genetic differences in the development of subsets of teeth [67]. A thorough assessment of these different possibilities will be required to fully understand how the network identified here applies to molars.

Further, what is the relationship between signaling pathways, core TF expression, and downstream TF output during dental lamina formation? Two major approaches that will be critical in defining these relationships include signaling pathway perturbation and molecular analysis of direct versus indirect effects. First, both genetic and pharmacological manipulation of signaling pathways in vivo or ex vivo will provide crucial insights into the initial establishment of domain specific TFs. Second, direct binding assays (e.g., CUT&RUN analysis) of both downstream outputs of signaling pathways (e.g., ß-catenin, LEF1, phospho-SMAD, etc.) and domain specific TFs (e.g., TFAP2A, TFAP2B, PITX2, SOX2) within relevant tissues (e.g., E10.5 orofacial epithelium) will narrow down key non-coding elements responsible for orchestrating these responses.

Finally, what are the causal mechanisms responsible for converting broad domains established by signaling molecules into sharp and precise boundaries? While our study suggests this is mediated by cross-repression between key groups of TFs, notably TFAP2 and PITX2, these interactions, and their molecular underpinnings, remain to be fully resolved. However, two mechanisms that likely account for this cross-repression include regulation at both transcriptional (i.e., non-coding cis-regulatory elements) and protein levels. In support of the former, we identified a highly conserved cis-regulatory elements near *Pitx2* and *Fgf8* loci with ATAC-seq data from our previous study [61]. Interestingly, accessibility at these elements negatively correlated with *Pitx2* and *Fgf8* gene expression and accessibility at these elements are decreased in *Tfap2a; Tfap2b EDKO’s*, suggesting that *Tfap2a/Tfap2b* might repress *Pitx2* at the transcriptional level (directly, or indirectly through regulation of FGF8). Conversely, in vitro luciferase reporter assays identified that TFAP2 can repress PITX2’s activation of a PITX2 responsive element (**S8C Fig**). Coupled with TFAP2A: PITX2 co-immunoprecipitation (Co-IP) experiment (**S8D Fig**), these data are consistent with potential direct protein-protein interaction in mediating repression. Whether similar mechanisms are deployed by PITX2 to counter a TFAP2-induced skin program will require additional studies. Ultimately, determining the molecular mechanisms of the cross-repression between domain specific TFs, including *Pitx2* and *Tfap2a/Tfap2b*, will provide a genetic blueprint for future regenerative strategies.

## Materials and methods

### Ethics statement

Mouse maintenance and mouse-related procedures were performed following protocols approved by the Institutional Animal Care and Use Committee of the University of Iowa.

### Mouse procedures and mouse lines

Embryos were staged by checking for vaginal plugs in the crossed females, with noon on the day a copulatory plug was present denoted as E0.5. Littermate embryos were used when comparing between genotypes. Yolk sacs or tail clips were used for genotyping of embryos. The *Pitx2* general knockout line (*Pitx2 KO*) has been previously described [15,68]. The *Tfap2a* conditional allele (*Tfap2a*^*flox*^) and *Tfap2b* conditional allele (*Tfap2b*^*flox*^) have been described previously [61,69–71]. To conditionally knockout *Tfap2a* and *Tfap2b* in mandibular epithelium starting around E7.5, we utilized the early ectodermal Cre line, CRECT, which is driven by a *Tfap2a* intronic ectodermal specific enhancer [52]. To monitor Cre recombination activity, we utilized the *Rosa26-mTmG* reporter line [72] (Jax Strain #:007576).

### Histology, H&E and immunofluorescence staining

Mouse embryos were harvested and washed with ice-cold 1× phosphate-buffered saline (PBS) and fixed with 4% paraformaldehyde in PBS for 4 hours. Samples for LMD-RNA-seq were fixed with methacarn (60% (vol/vol) absolute methanol, 30% chloroform, and 10% glacial acetic acid) for 4 hours. Following fixation, samples were dehydrated through a graded ethanol series, embedded in paraffin and sectioned (7 μm). Standard Hematoxylin and Eosin (H&E) staining was used to examine tissue morphology. For immunohistochemistry and immunofluorescence, sections were incubated in 0.01 M citrate buffer (pH = 6.0) in a 100°C water bath for 20 min or autoclaved in 0.1 M Tris-HCl buffer (pH 9.0) for 5 minutes. Sections were then blocked with 20% donkey serum and incubated with primary antibodies overnight at 4°C. Primary antibodies (αTFAP2A, DSHB, 3B5; αTFAP2B, Santa Cruz, sc-8976, Cell Signaling, 2509; αLEF1, Cell Signaling, 2230; FoxF1, R&D Systems, AF4798; αSOX2, R&D Systems, AF2018, Abcam, ab92494; αPITX1, Novusbio, NBP1-88644; and αPITX2, R&D Systems, AF7388) were diluted in PBS containing 1% donkey serum. Then sections were washed with PBST (0.05% Tween-20 in PBS), incubated with secondary antibodies, and stained with DAPI. Images were captured using a Nikon Eclipse microscope or a Zeiss 700 confocal microscope.

### *in situ* hybridization (ISH)

Mouse embryos were fixed in 4% paraformaldehyde (PFA) at 4°C for 24 hours, dehydrated in 30% sucrose/PBS at 4°C overnight, and cryoembedded in optimal cutting temperature (OCT) embedding medium. 10-μm-thick frozen sections were cut and preserved at -80°C before *in situ* hybridization. A single molecule fluorescence *in situ* hybridization method named PLISH was performed as previously described [73] with some modifications [74]. Briefly, frozen sections were post-fixed in cold 4% PFA for 20 minutes, incubated in citrate-based target unmasking solution (Vector laboratories, H-3300, Burlingame, CA) with 0.05% lithium dodecyl sulfate (Sigma, L4632, St. Louis, MO) at 65°C for 30 minutes. The slides were then incubated with freshly prepared 0.05 mg/ml pepsin (Sigma-Aldrich, P6887, St. Louis, MO) in 0.1 M HCl for 10 minutes at 37°C. Next, tissue sections were sequentially incubated with short-paired hybridization probes for Pitx2 and Lef-1 (2 hours), circle and bridge probes (1 hour), T4 DNA ligase mixture (ligation, 2 hours), DNA polymerase mixture (rolling circle amplification, 4 hours), and fluorescence labeling probes (30 minutes). Sections were briefly washed, mounted in anti-fade mounting medium with DAPI (Vector Laboratories, H-1500), and imaged with a 60x/1.42 oil lens using the Olympus FV3000 Confocal Laser Scanning Microscope.

RNA sequences that were targeted by short-paired hybridization probes were:

Mouse *Pitx2*-1: 5’-CGCGCCGCCGACTCCTCCGTACGTTTATAGGGACACATGT-3’;

Mouse *Pitx2*-2: 5’-GAAAGACTGAGAATTGTGCTAGAAGGTCGTGCGCACTATG-3’;

Mouse *Pitx2*-3: 5’-AGGAATGTCCCCAAGTGTCTACGTCTTTCGCTAAGAGTAT-3’;

Mouse *Pitx2*-4: 5’-CCACAAATGTTTGACTGGATATGACATTTTAACATTACTA-3’;

Mouse *Lef1*-1: 5’-GTGTGTGTGTAAGTGTGTGTATCGGCCCGAGCTGCGGGCT-3’;

Mouse *Lef1*-2: 5’-AAGGGAAGGAAAGAAGCTCTAACGCGGACGTCTGCAGCCC-3’;

Mouse *Lef1*-3: 5’-CAGCCCTGGCAGCCTAGCCTAGTGCACGCGGAGCGGCGTA-3’;

Mouse *Lef1*-4: 5’-CCCGCAGCGGAGCGGAGATTACACAGCCGCCGGGATGCCC-3’;

### Laser Microdissection (LMD) coupled RNA-seq

Laser Microdissection was performed with the Leica LMD7000 Laser Microdissection System. For tissue fixation, Methacarn solution was used. Tissues were then dehydrated through a graded ethanol series, embedded in paraffin wax and sectioned (7 μm). Selected regions of tissue sections were cut at 20× magnification while keeping laser power to a minimum. At least 3 embryos per genotype were used to collect tissues for LMD-RNA-seq. After cells were collected in cap, tubes were spun down quickly and 5 μl of Smart-seq2 lysis buffer (with 0.5% Triton X-100, 5μg Proteinase K and 2 U μl^−1^ RNase inhibitor) was added, followed by pipetting up and down to mix. Cells were lysed for 1 hour at 60°C. Then samples were treated with 1U DNase I for 30 min. The standard Smart-seq2 protocol was followed for cDNA preparation, Tn5 Tagmentation, and library preparation for next generation sequencing. Quality of cDNA and sequencing libraries were determined using a Bioanalyzer system (Agilent) and real time PCR. The KAPA Library Quantification Kit (Roche) was used for quantification of RNA sequencing libraries.

### RNA-seq data analysis

RNA-seq reads were quality checked using the FastQC tool (http://www.bioinformatics.babraham.ac.uk/projects/fastqc). Low-quality and adapter sequences were removed using Trimmomatic [75]. Expression of transcripts was quantified using Salmon [76], and estimates of transcript abundance for gene-level analysis were imported and summarized using the tximport [77] function of the R/Bioconductor software suite [78]. Differentially expressed genes (DEGs) were identified by applying the R/Bioconductor package DeSeq2[33]. To control for variability associated with lowly expressed genes, we further applied the ASHR algorithm [34] to estimate shrunken (conservative) log2 fold-change. We use following threshold to identify DEGs: absolute log2 fold-change larger than 1.0 and adjusted p value less than 0.01. We use Heatmaps were generated with Morpheus (https://software.broadinstitute.org/morpheus).

### Cloning, transient transfection, luciferase assay, RT-PCR and western blotting

A 1.6 kilobase (kb) fragment, encompassing the *Pitx2* promoter and including an auto-regulatory PITX2-binding site, was cloned and inserted into the pGL4 luciferase reporter vector. A modified sequence- and ligation-independent cloning (SLIC)[79] protocol was used for cloning. GMSM-K[53] cells, a human oral epithelial cell line, were seeded in flasks or plates in Dulbecco’s Modified Eagle’s Medium (DMEM) with 10% fetal bovine serum, and fed at least 24 hours prior to the experiments. GMSM-K cells were transfected with DNA constructs (total 1 μg/well in a 12-well plate) using Lipofectamine 2000 reagent (Invitrogen, 11668019, Calsbad, CA) in Opti-MEM medium (Thermo Fisher Scientific, 31985088, Waltham, MA). At 48 hours post transfection, the activities of Firefly luciferase and β-galactosidase activity were measured using the Luciferase Assay System (Promega, E1500, Madison, WI) and β-galactosidase Assay System (Galacto-Light Plus reagents, Tropix Inc), separately according to the methods previously described [80]. For quantitative real-time RT-PCR, total RNA was isolated using the Trizol reagent (Invitrogen, 15596026, Carlsbad, CA) and reverse transcribed into cDNA using a PrimeScript RT Master Mix (Takara RR036A, Kusatsu, Shiga, Japan). Quantitative real-time RT-PCR analysis was performed using TB Green Premix Ex Taq (Clontech, RR420A, Mountain View, CA) and following primers: ACTB-F:CTCTTCCAGCCTTCCTTC; ACTB-R:ATCTCCTTCTGCATCCTGTC; Hprt-F:CAGTCCCAGCGTCGTGATTA; Hprt-R:GGCCTCCCATCTCCTTCATG; TFAP2A-rtF1,GACCTCTCGATCCACTCCTTAC; TFAP2A-rtR1,GAGACGGCATTGCTGTTGGACT. Statistical differences of numeric parameters between two groups were determined with one-way ANOVA. The statistical analyses were performed in Prism. For western blotting, cells were lysed with a lysis buffer containing 1% of NP-40, 0.1% SDS, 0.25% deoxycholate and protease inhibitors cocktail (Sigma-Aldrich, P8340, St. Louis, MO). Protein concentration was determined by Pierce BCA kit (Thermo Fisher Scientific, 23225, Waltham, MA) and samples were subjected to SDS-polyacrylamide gel electrophoresis. Membranes were immunoblotted with anti-TFAP2A (DSHB, 3B5) at a 1:500 dilution. Signal was detected using the Clarity Western ECL Blotting Substrate (BioRad, Hercules, CA).

## Supporting information

S1 Fig Pairwise differentially expressed genes analysis of the E11.5 mouse mandibular epithelium along the dorsal-ventral axis. **A-E)** Volcano plot showing pairwise differentially expressed genes (DEGs) analysis of 4 different domains (as shown in Fig 1B) of mouse E11.5 mandibular epithelium along the dorsoventral axis at future incisor site (medial). **F)** Comparing dental lamina and aboral epithelium at future molar site (lateral) of mouse E11.5 mandibular epithelium. Red dots indicate DEGs with a absolute log2 fold-change larger than 1.0 and a adjusted p value less than 0.01.
(TIF)

S2 Fig Expression heatmap of domain specific and domain enriched transcription factors. **A)** Expression heatmap of domain specific and domain enriched transcription factors, including those not presented in **Fig 2B**, along the dorsoventral axis of the E11.5 mandibular epithelium. Rows represent genes, columns represent samples. log2(TPM+1) expression values were used in the heatmap. Abbreviation: TPM, Transcript Per Million.
(TIF)

S3 Fig Expression patterns of domain specific transcription factors. **A, B, C**) SOX2, TFAP2B and merged immunofluorescent (IF) staining of an E9.5 mouse head sagittal section. **D, E, F**) SOX2, LEF1 and merged IF staining of an E9.5 mouse head sagittal section. **G, H, I**) PITX2, PITX1 and merged IF staining of an E11.5 mouse head sagittal section. **J, K, L**) PITX2, LEF1 and merged IF staining (protein level) of an E11.5 mouse head sagittal section. **M, N, O**) *Pitx2*, *Lef1* and merged ISH staining (mRNA level) of an E11.5 mouse head sagittal section. White arrowheads in all panels point to the dental lamina. Abbreviations: DAPI, DAPI nuclear counterstain; DL, dental lamina; Md, Mandible; Mx, Maxilla. Scale bar for panels A-C; 100 μm; Scale bar for panels D-F: 20 μm; Scale bar for panels G-O: 100 μm.
(TIF)

S4 Fig Enriched signaling pathway genes along the mandibular epithelial dorsoventral axis. **A)** Expression heatmap of differentially expressed signaling pathway genes associated with the 4 domains profiled along the dorsoventral axis of the mouse E11.5 mandibular epithelium. Rows represent genes, columns represent samples. log2(TPM+1) expression values were used in the heatmap. Abbreviations: TPM, transcript per million.
(TIF)

S5 Fig Tooth development arrest in *Pitx2 KO* and formation of the aboral ectopic incisor in the *Tfap2a; Tfap2b EDKO*. **A-F)** H&E staining of a sagittal section of an E9.5; E11.5; and E12.5 control and *Pitx2 KO* head. Black dashed lines outline the dental lamina and dental placode**. G, H)** H&E staining of a sagittal section of an E12.5 control and *Tfap2a; Tfap2b EDKO* head. H’: Higher magnification of boxed region in panel H. Black dashed lines outline the placode of the first incisor (I_1_) and ectopic incisor (EI) in *Tfap2a; Tfap2b EDKO*s. **I, J)** H&E staining of a sagittal section of an E14.5 control and *Tfap2a; Tfap2b EDKO* head. I’: Higher magnification of boxed region in panel I. J’: Higher magnification of boxed region in panel J. Black and red dashed lines outline the first incisor (I_1_) and ectopic incisor (EI), respectively, in *Tfap2a; Tfap2b EDKO*s. **K)** A table summarizing the penetrance of the ectopic incisor phenotype in *Tfap2a; Tfap2b EDKOs* at different developmental stages. H&E refers to visualization by H&E staining of sagittal sections, like in panels G-J, whereas ’skeleton’ refers to visualization by bone and cartilage staining of the craniofacial skeleton. Abbreviations: EI, ectopic incisor; I_1_, incisor at original site; M, molar; Md, mandible; T, tongue.
(TIF)

S6 Fig Reverse labial-lingual axis of the ectopic incisor in the *Tfap2a; Tfap2b EDKO*. A, B) H&E staining of a sagittal section of an E14.5 control and *Tfap2a; Tfap2b EDKO* mandible. For *Tfap2a; Tfap2b EDKO* mandible, medial to lateral sections reveal the labial-lingual axis of the ectopic incisor is reversed. Abbreviations: EI, ectopic incisor; I_1_, incisor at original site. Scale bar = 100 μm.
(TIF)

S7 Fig No change of dental lamina specific genes in single KO of *Tfap2a* or *Tfap2b*. **A-C)** SOX2 immunofluorescent staining of an E11.5 mouse head sagittal section in a control; *Tfap2a single KO* and *Tfap2b single KO*. **D-F)** PITX2 immunofluorescent staining of an E11.5 mouse head sagittal section in a control; *Tfap2a single KO* and *Tfap2b single KO*. White arrowheads in all panels point to the dental lamina. Abbreviations: DAPI, DAPI nuclear counterstain; DL, dental lamina; Md, Mandible; Mx, Maxilla. Scale bar = 100 μm.
(TIF)

S8 Fig Cross-group repression between *Tfap2* and *Pitx2*. **A)** TFAP2A mRNA relative expression measured by real-time qPCR in GMSMK cells transfected with GFP or *Pitx2* plasmids. **B)** Western blot of TFAP2A in GMSMK cells transfected with GFP or *Pitx2* plasmids. **C)** Tfap2a repress Pitx2’s activation of Pitx2 promoter in dose responsive manner. **D)** Western blot of TFAP2A after Co-immunoprecipitation (Co-IP) with PITX2 or IgG antibody in HEK293T cells transfected with the indicated constructs. * means p value less than 0.05; **** means p value less than 0.0001.
(TIF)

S9 Fig SHH and WNT signaling pathways form complementary expression domains in the dental mesenchyme during tooth initiation. **A)** A volcano plot of DEGs comparing the E11.5 WT mesenchyme on the posterior or anterior side of the dental lamina. Red dots indicate DEGs with a absolute log2 fold-change larger than 1.0 and a adjusted p value less than 0.01. **B)** FOXF1 (top, red) or LEF1 (middle, green) immunofluorescent (IF) staining of an E10.5 mouse head sagittal section. Note, the bottom panel includes visualization of both channels along with the DAPI counterstain. Abbreviations: 2^nd^, second brachial arch; DAPI, DAPI nuclear counterstain; DL, dental lamina; Md, Mandible. Scale bar = 100 μm.
(TIF)

S10 Fig Dysregulation of SHH and WNT pathway activity in the dental mesenchyme of *Pitx2 KOs*. **A-F)** FOXF1 (A, D) or LEF1 (B, E) IF staining of an E11.5 mouse head sagittal section in a control (A-C) or *Pitx2 KO* (D-F). Note, panels C and F are the same section as in panels A, B or D, E, respectively, but include both channels and visualization of the DAPI counterstain. The white arrowhead in all panels point to the dental lamina. **C’** and **F’** show higher magnification of the dental lamina from panels **C** and **F**. The three white arrowheads highlight the reduction of FOXF1 and upregulation of LEF1 in the posterior dental mesenchyme of *Pitx2 KOs* (F’), relative to controls (C’). Abbreviations: DAPI, DAPI nuclear counterstain; DL, dental lamina; Md, mandible; Mx, maxillary. Scale bar = 100 μm.
(TIF)

S11 Fig Expansion and upregulation of WNT signal in *Pitx2 KOs*. **A, C)** LEF1 immunofluorescent staining of an E11.5 mouse head sagittal section in a control and *Pitx2 KOs*. **A’, C’**: higher magnification of dental lamina region in panel **A** and **C**. **B, D)** CTNNB1 (β-catenin) immunofluorescent staining of an E11.5 mouse head sagittal section in a control and *Pitx2 KOs*. **B’, D’**: higher magnification of dental lamina region in panel **B** and **D**. Three arrows point to posterior side of the dental mesenchyme.
(TIF)

S1 Table List of domain specific or enriched genes along the dorsal-ventral axis of E11.5 mouse mandibular epithelium. Columns A-C: domain specific genes for dorsal/posterior domain epithelium; dental lamina; and ventral/aboral domain epithelium. Columns D-F: domain enriched genes for dorsal/posterior domain epithelium; dental lamina; and ventral/aboral domain epithelium.
(XLSX)
