# Supplementary figures and images for: Transcriptional programs of Pitx2 and Tfap2a/Tfap2b controlling lineage specification of mandibular epithelium during tooth initiation
Source: PLoS Genet. 2024 Jul 25;20(7):e1011364. doi: 10.1371/journal.pgen.1011364 (PMC11302917; doi:10.1371/journal.pgen.1011364)

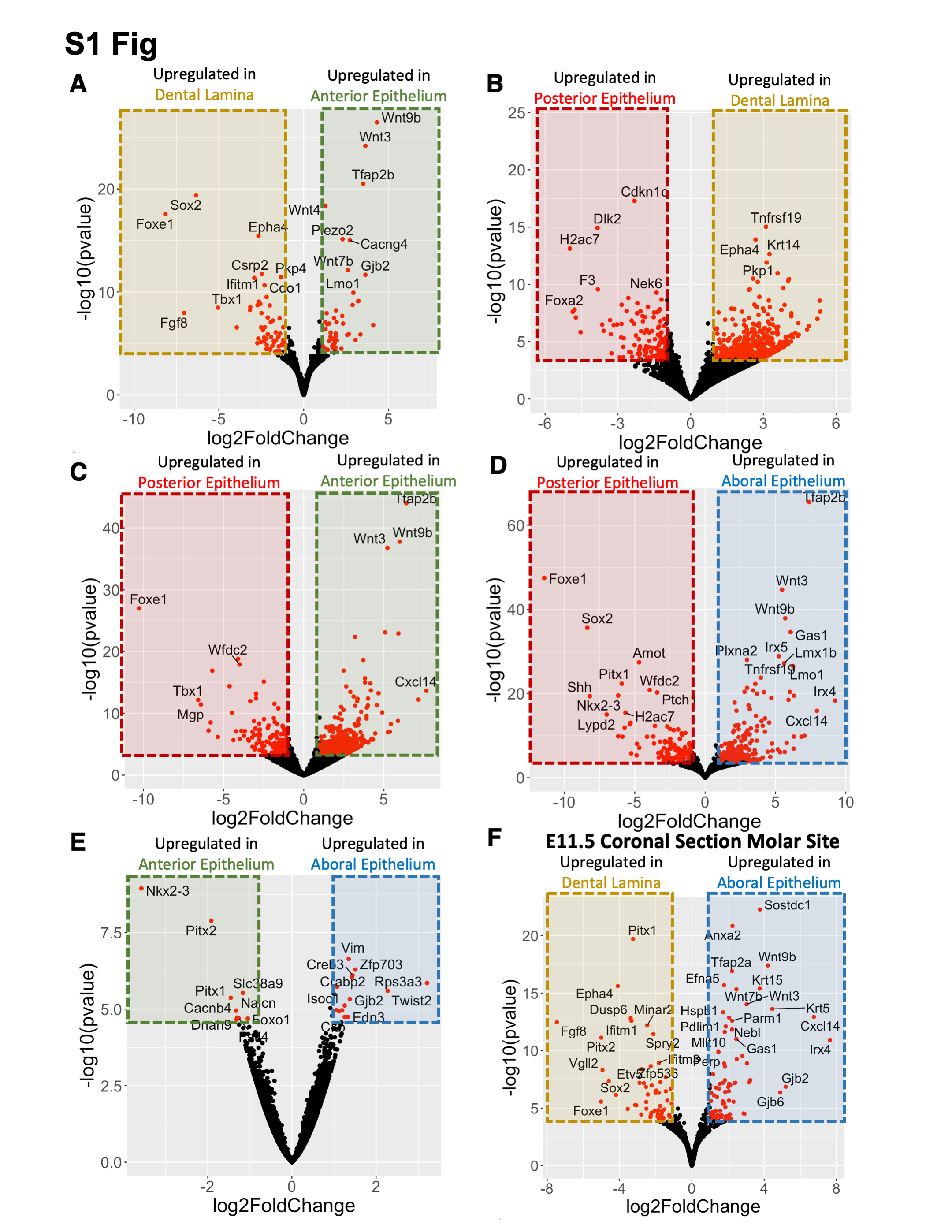

Supplement: S1 Fig — A-E) Volcano plot showing pairwise differentially expressed genes (DEGs) analysis of 4 different domains (as shown in Fig 1B) of mouse E11.5 mandibular epithelium along the dorsoventral axis at future incisor site (medial). F) Comparing dental lamina and aboral epithelium at future molar site (lateral) of mouse E11.5 mandibular epithelium. Red dots indicate DEGs with a absolute log2 fold-change larger than 1.0 and a adjusted p value less than 0.01. (TIF) [file pgen.1011364.s001.tif]

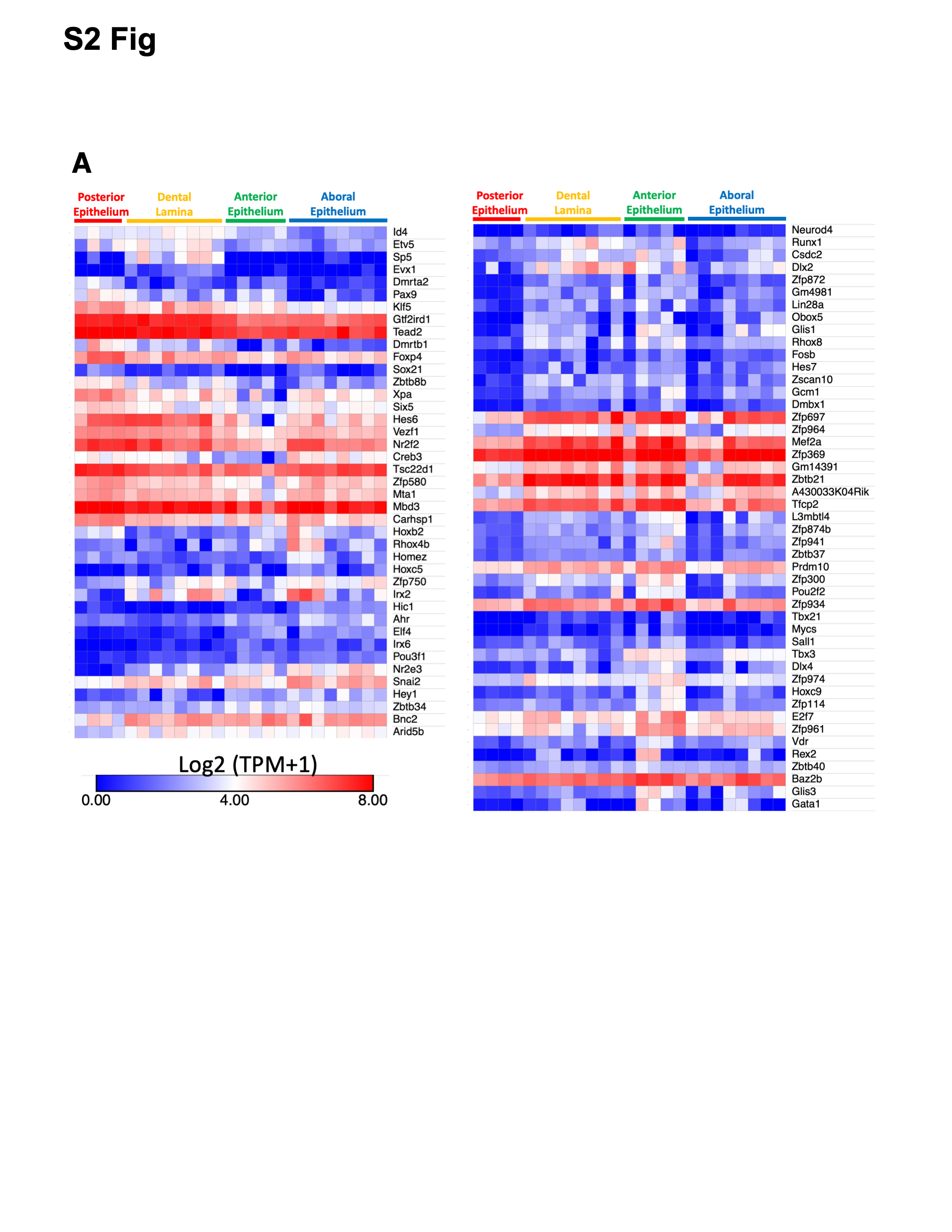

Supplement: S2 Fig — A) Expression heatmap of domain specific and domain enriched transcription factors, including those not presented in Fig 2B, along the dorsoventral axis of the E11.5 mandibular epithelium. Rows represent genes, columns represent samples. log2(TPM+1) expression values were used in the heatmap. Abbreviation: TPM, Transcript Per Million. (TIF) [file pgen.1011364.s002.tif]

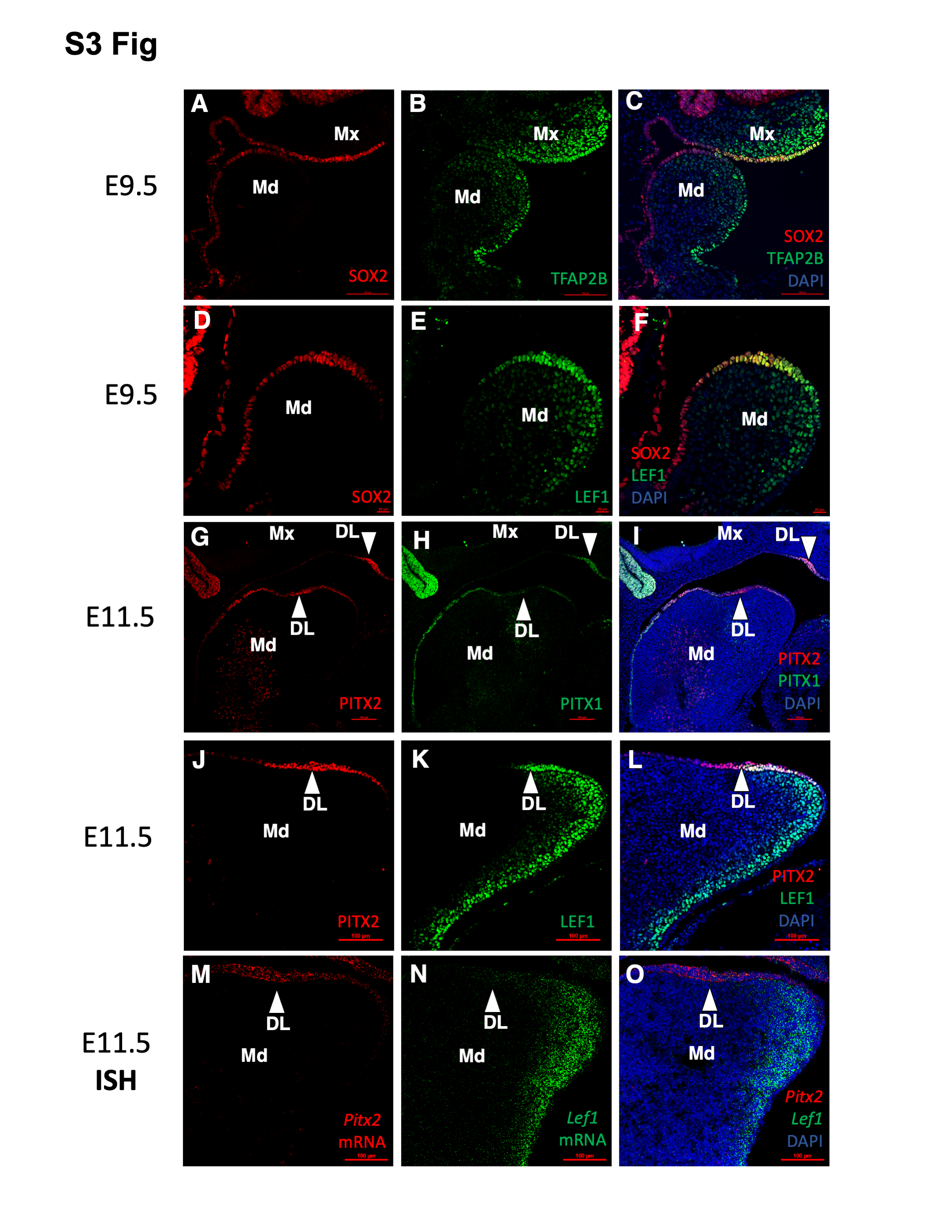

Supplement: S3 Fig — A, B, C) SOX2, TFAP2B and merged immunofluorescent (IF) staining of an E9.5 mouse head sagittal section. D, E, F) SOX2, LEF1 and merged IF staining of an E9.5 mouse head sagittal section. G, H, I) PITX2, PITX1 and merged IF staining of an E11.5 mouse head sagittal section. J, K, L) PITX2, LEF1 and merged IF staining (protein level) of an E11.5 mouse head sagittal section. M, N, O) Pitx2, Lef1 and merged ISH staining (mRNA level) of an E11.5 mouse head sagittal section. White arrowheads in all panels point to the dental lamina. Abbreviations: DAPI, DAPI nuclear counterstain; DL, dental lamina; Md, Mandible; Mx, Maxilla. Scale bar for panels A-C; 100 μm; Scale bar for panels D-F: 20 μm; Scale bar for panels G-O: 100 μm. (TIF) [file pgen.1011364.s003.tif]

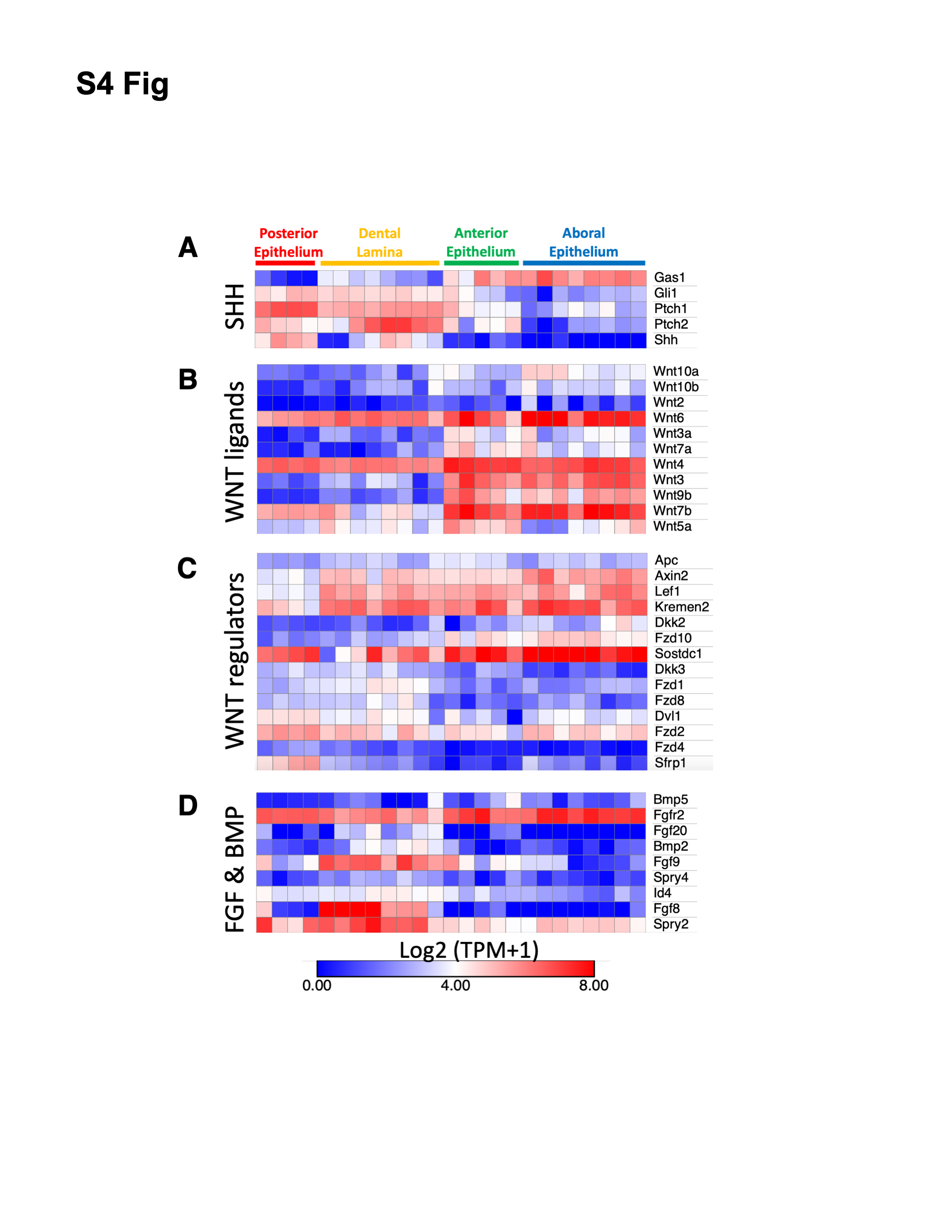

Supplement: S4 Fig — A) Expression heatmap of differentially expressed signaling pathway genes associated with the 4 domains profiled along the dorsoventral axis of the mouse E11.5 mandibular epithelium. Rows represent genes, columns represent samples. log2(TPM+1) expression values were used in the heatmap. Abbreviations: TPM, transcript per million. (TIF) [file pgen.1011364.s004.tif]

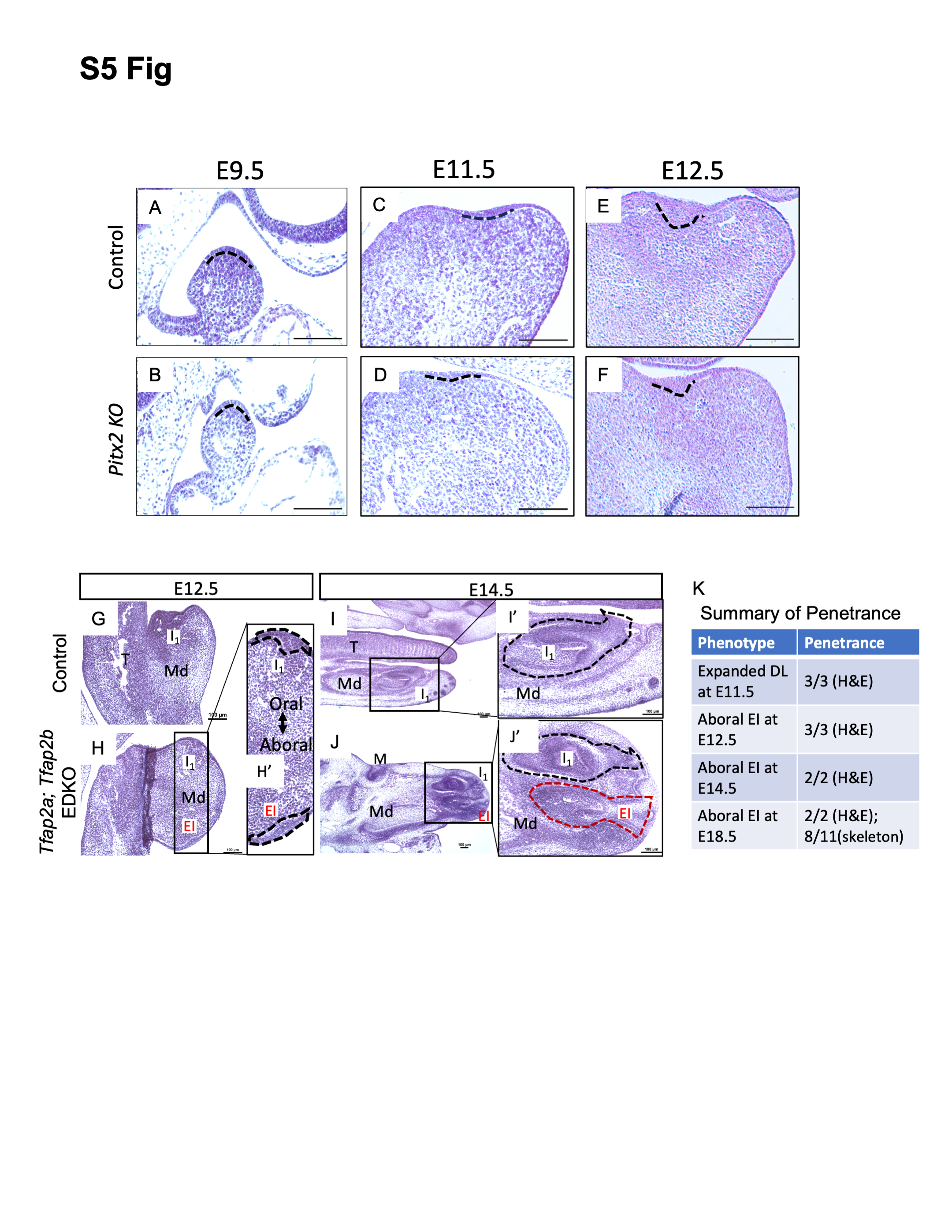

Supplement: S5 Fig — A-F) H&E staining of a sagittal section of an E9.5; E11.5; and E12.5 control and Pitx2 KO head. Black dashed lines outline the dental lamina and dental placode. G, H) H&E staining of a sagittal section of an E12.5 control and Tfap2a; Tfap2b EDKO head. H’: Higher magnification of boxed region in panel H. Black dashed lines outline the placode of the first incisor (I1) and ectopic incisor (EI) in Tfap2a; Tfap2b EDKOs. I, J) H&E staining of a sagittal section of an E14.5 control and Tfap2a; Tfap2b EDKO head. I’: Higher magnification of boxed region in panel I. J’: Higher magnification of boxed region in panel J. Black and red dashed lines outline the first incisor (I1) and ectopic incisor (EI), respectively, in Tfap2a; Tfap2b EDKOs. K) A table summarizing the penetrance of the ectopic incisor phenotype in Tfap2a; Tfap2b EDKOs at different developmental stages. H&E refers to visualization by H&E staining of sagittal sections, like in panels G-J, whereas ’skeleton’ refers to visualization by bone and cartilage staining of the craniofacial skeleton. Abbreviations: EI, ectopic incisor; I1, incisor at original site; M, molar; Md, mandible; T, tongue. (TIF) [file pgen.1011364.s005.tif]

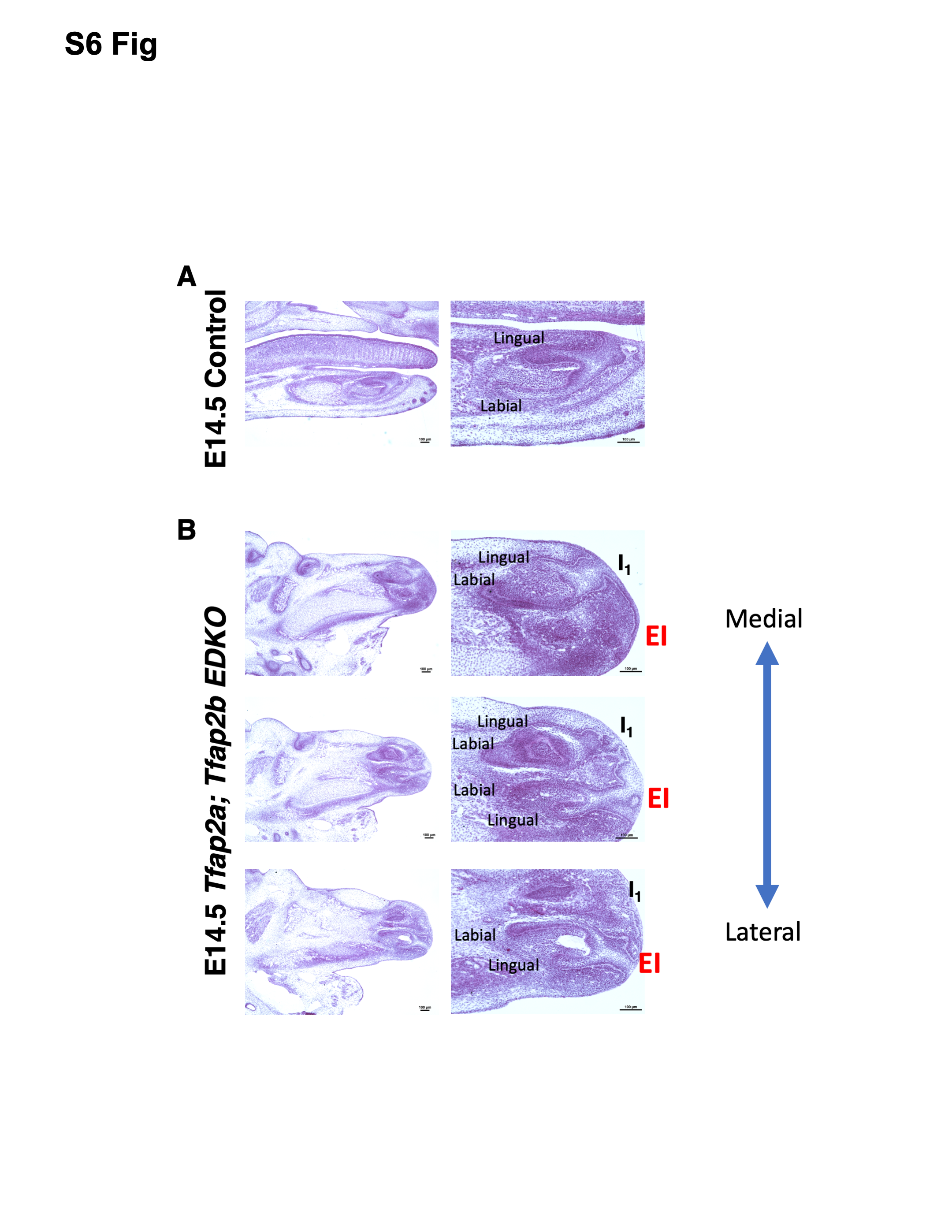

Supplement: S6 Fig — A, B) H&E staining of a sagittal section of an E14.5 control and Tfap2a; Tfap2b EDKO mandible. For Tfap2a; Tfap2b EDKO mandible, medial to lateral sections reveal the labial-lingual axis of the ectopic incisor is reversed. Abbreviations: EI, ectopic incisor; I1, incisor at original site. Scale bar = 100 μm. (TIF) [file pgen.1011364.s006.tif]

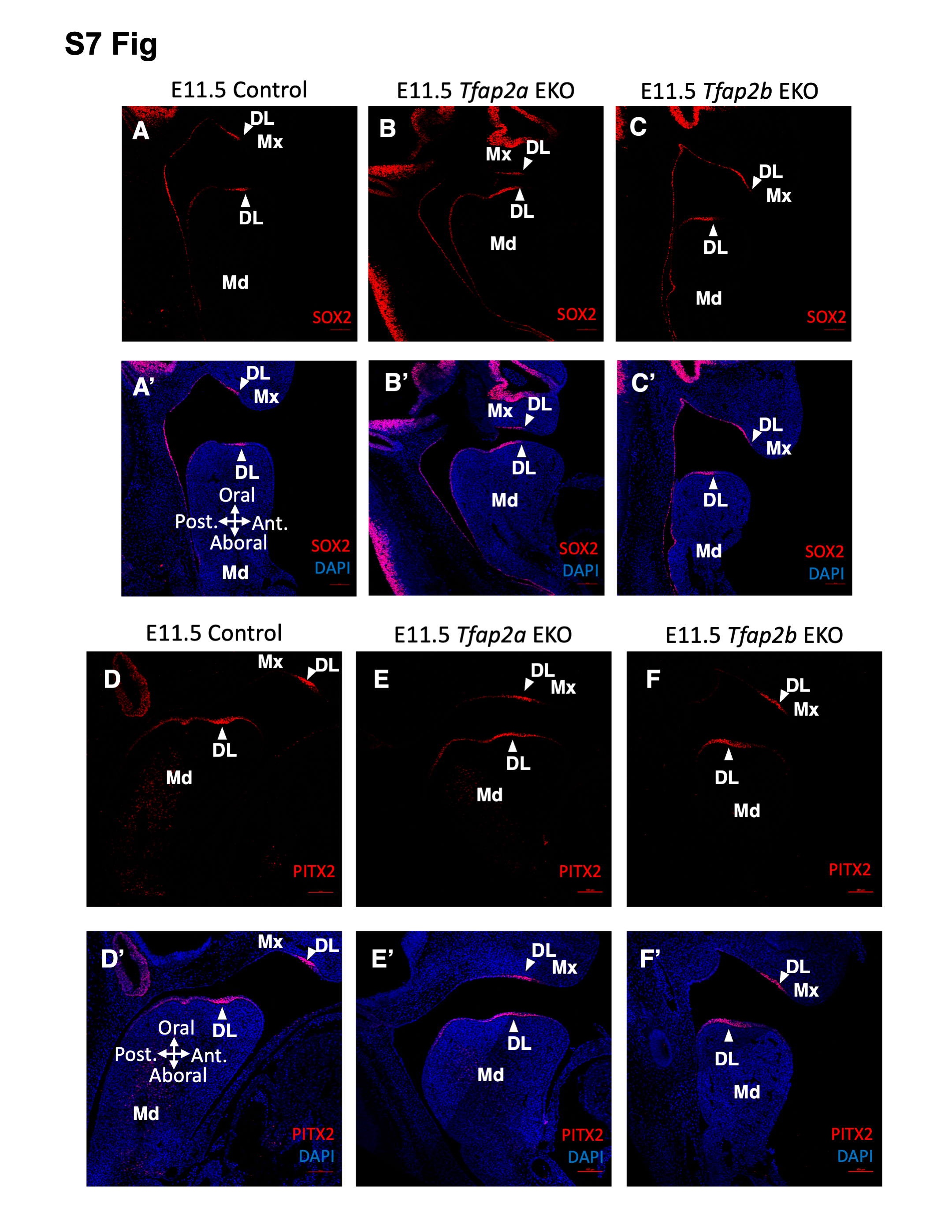

Supplement: S7 Fig — A-C) SOX2 immunofluorescent staining of an E11.5 mouse head sagittal section in a control; Tfap2a single KO and Tfap2b single KO. D-F) PITX2 immunofluorescent staining of an E11.5 mouse head sagittal section in a control; Tfap2a single KO and Tfap2b single KO. White arrowheads in all panels point to the dental lamina. Abbreviations: DAPI, DAPI nuclear counterstain; DL, dental lamina; Md, Mandible; Mx, Maxilla. Scale bar = 100 μm. (TIF) [file pgen.1011364.s007.tif]

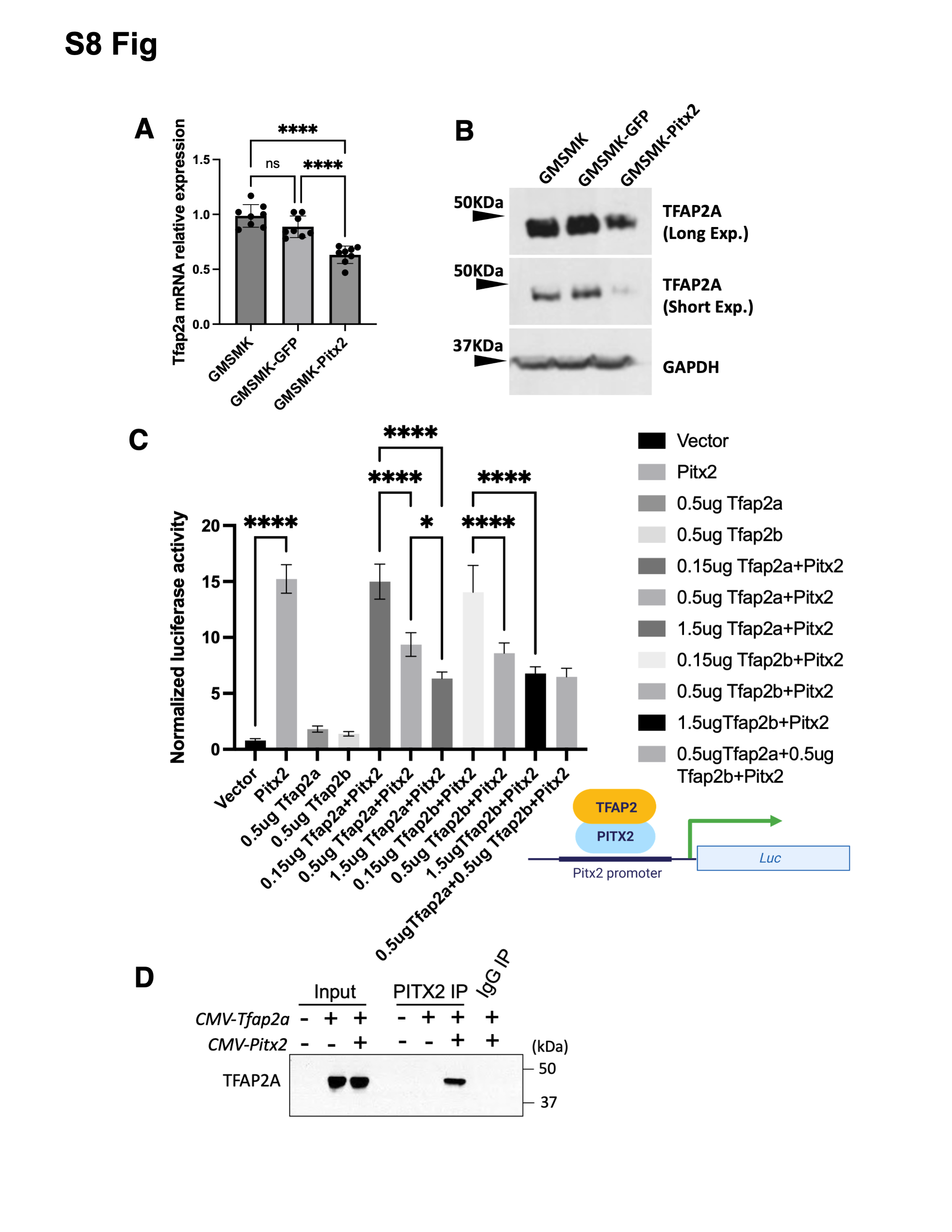

Supplement: S8 Fig — A) TFAP2A mRNA relative expression measured by real-time qPCR in GMSMK cells transfected with GFP or Pitx2 plasmids. B) Western blot of TFAP2A in GMSMK cells transfected with GFP or Pitx2 plasmids. C) Tfap2a repress Pitx2’s activation of Pitx2 promoter in dose responsive manner. D) Western blot of TFAP2A after Co-immunoprecipitation (Co-IP) with PITX2 or IgG antibody in HEK293T cells transfected with the indicated constructs. * means p value less than 0.05; **** means p value less than 0.0001. (TIF) [file pgen.1011364.s008.tif]

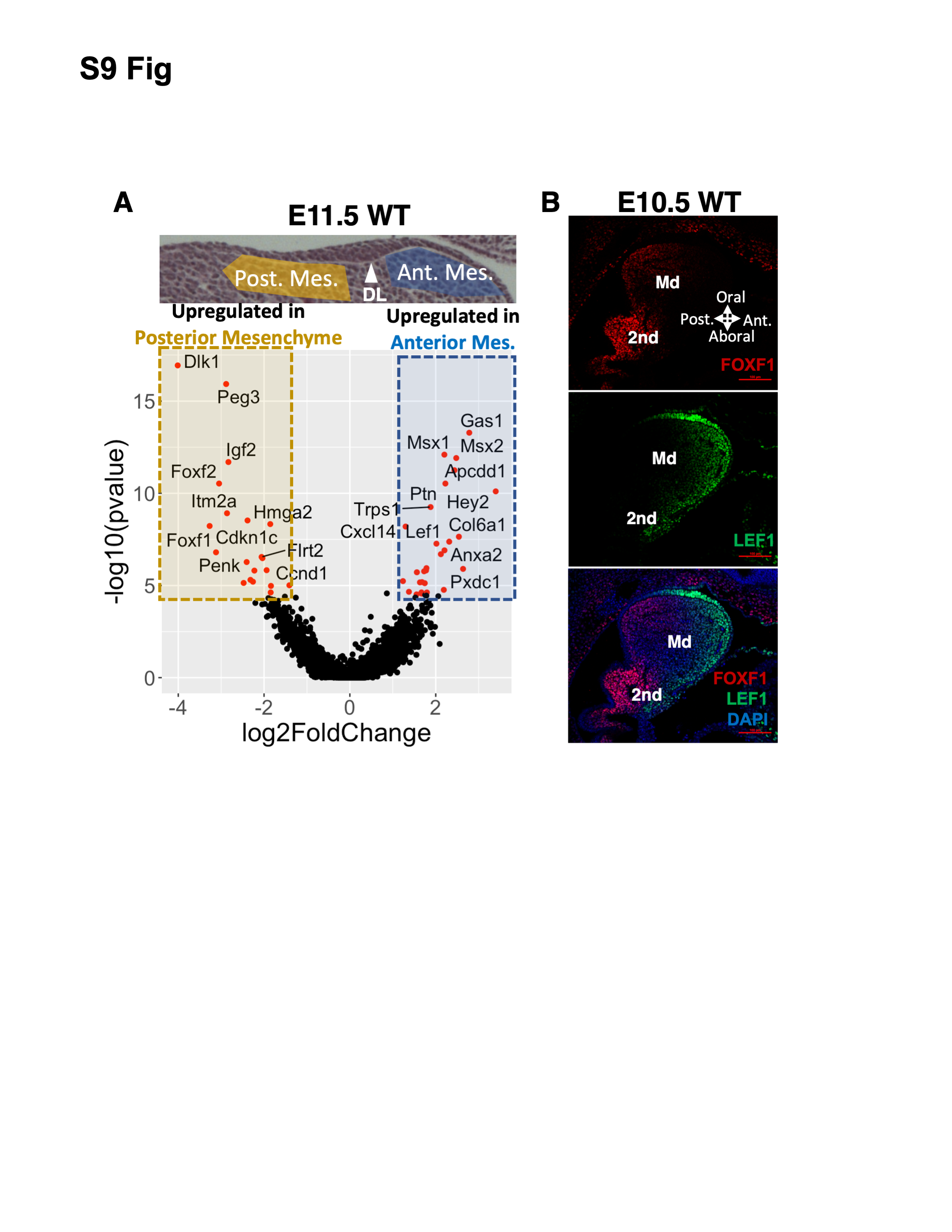

Supplement: S9 Fig — A) A volcano plot of DEGs comparing the E11.5 WT mesenchyme on the posterior or anterior side of the dental lamina. Red dots indicate DEGs with a absolute log2 fold-change larger than 1.0 and a adjusted p value less than 0.01. B) FOXF1 (top, red) or LEF1 (middle, green) immunofluorescent (IF) staining of an E10.5 mouse head sagittal section. Note, the bottom panel includes visualization of both channels along with the DAPI counterstain. Abbreviations: 2nd, second brachial arch; DAPI, DAPI nuclear counterstain; DL, dental lamina; Md, Mandible. Scale bar = 100 μm. (TIF) [file pgen.1011364.s009.tif]

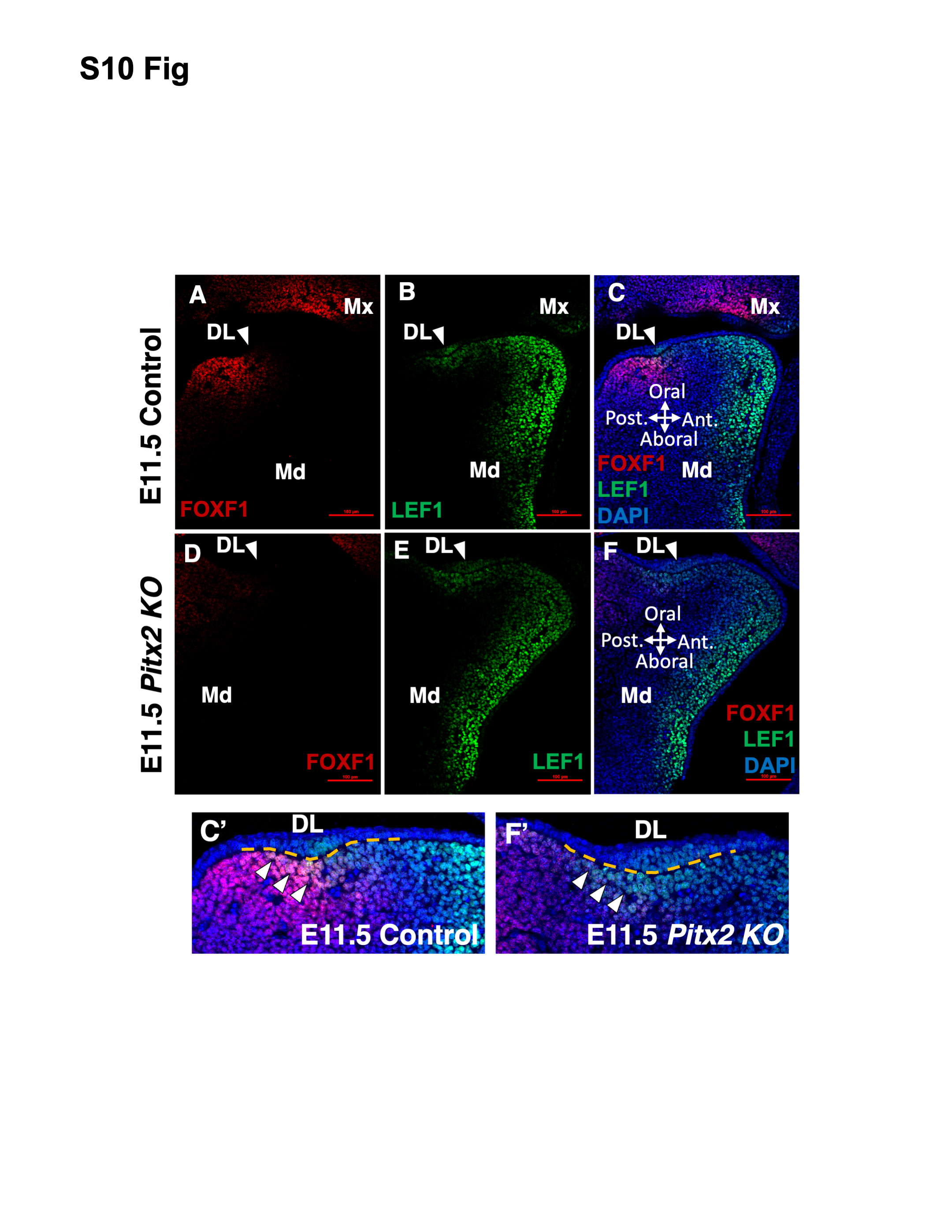

Supplement: S10 Fig — A-F) FOXF1 (A, D) or LEF1 (B, E) IF staining of an E11.5 mouse head sagittal section in a control (A-C) or Pitx2 KO (D-F). Note, panels C and F are the same section as in panels A, B or D, E, respectively, but include both channels and visualization of the DAPI counterstain. The white arrowhead in all panels point to the dental lamina. C’ and F’ show higher magnification of the dental lamina from panels C and F. The three white arrowheads highlight the reduction of FOXF1 and upregulation of LEF1 in the posterior dental mesenchyme of Pitx2 KOs (F’), relative to controls (C’). Abbreviations: DAPI, DAPI nuclear counterstain; DL, dental lamina; Md, mandible; Mx, maxillary. Scale bar = 100 μm. (TIF) [file pgen.1011364.s010.tif]

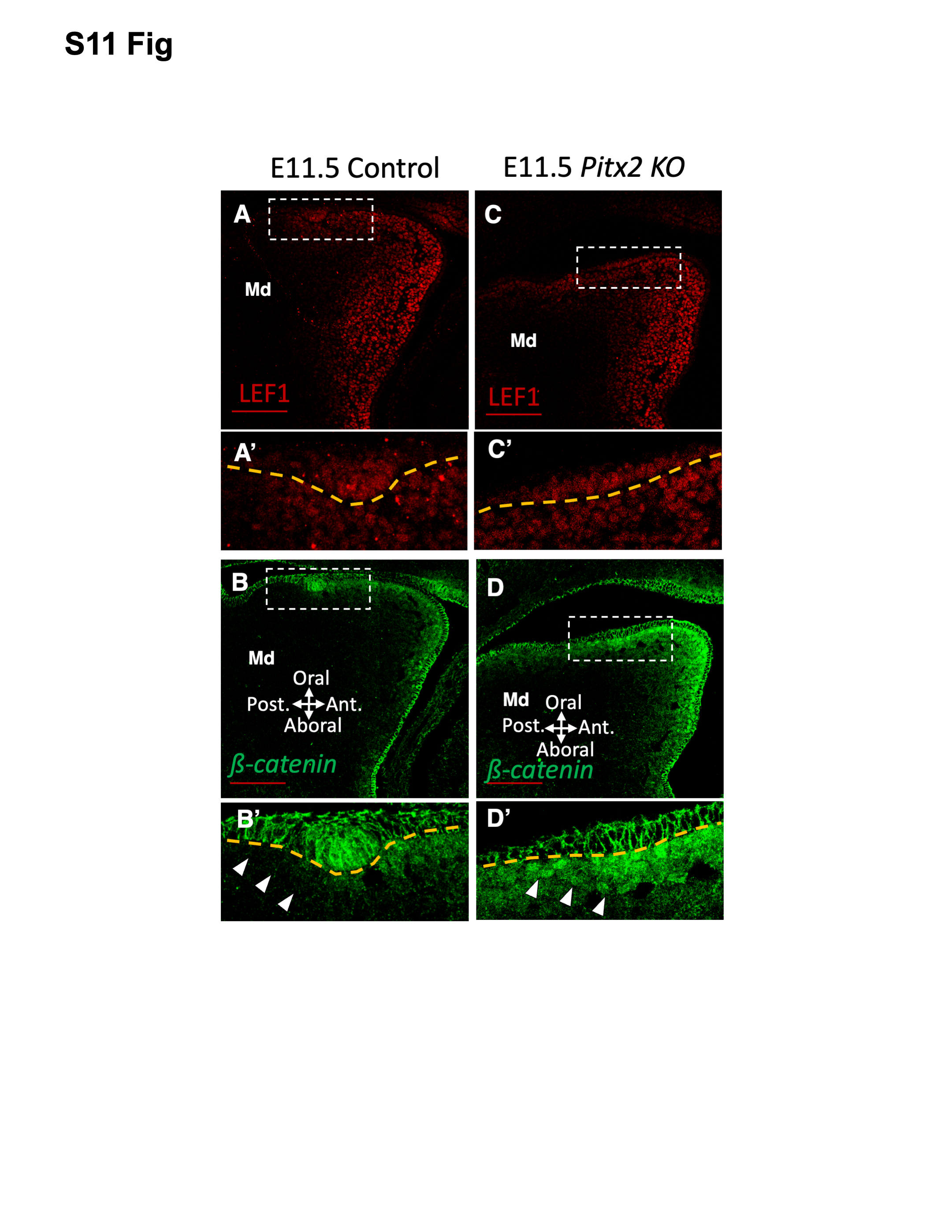

Supplement: S11 Fig — A, C) LEF1 immunofluorescent staining of an E11.5 mouse head sagittal section in a control and Pitx2 KOs. A’, C’: higher magnification of dental lamina region in panel A and C. B, D) CTNNB1 (β-catenin) immunofluorescent staining of an E11.5 mouse head sagittal section in a control and Pitx2 KOs. B’, D’: higher magnification of dental lamina region in panel B and D. Three arrows point to posterior side of the dental mesenchyme. (TIF) [file pgen.1011364.s011.tif]
